# Soil bacterial populations are shaped by recombination and gene-specific selection across a grassland meadow

**DOI:** 10.1038/s41396-020-0655-x

**Published:** 2020-04-23

**Authors:** Alexander Crits-Christoph, Matthew R. Olm, Spencer Diamond, Keith Bouma-Gregson, Jillian F. Banfield

**Affiliations:** 1 Department of Plant and Microbial Biology, University of California, Berkeley, CA USA; 2 Department of Earth and Planetary Science, University of California, Berkeley, CA USA; 3 Department of Environmental Science, Policy, and Management, University of California, Berkeley, CA USA; 4 Earth Sciences Division, Lawrence Berkeley National Laboratory, Berkeley, CA USA; 5 Chan Zuckerberg Biohub, San Francisco, CA USA

**Keywords:** Metagenomics, Population genetics, Microbial ecology

## Abstract

Soil microbial diversity is often studied from the perspective of community composition, but less is known about genetic heterogeneity within species. The relative impacts of clonal interference, gene-specific selection, and recombination in many abundant but rarely cultivated soil microbes remain unknown. Here we track genome-wide population genetic variation for 19 highly abundant bacterial species sampled from across a grassland meadow. Genomic inferences about population structure are made using the millions of sequencing reads that are assembled de novo into consensus genomes from metagenomes, as each read pair describes a short genomic sequence from a cell in each population. Genomic nucleotide identity of assembled genomes was significantly associated with local geography for over half of the populations studied, and for a majority of populations within-sample nucleotide diversity could often be as high as meadow-wide nucleotide diversity. Genes involved in metabolite biosynthesis and extracellular transport were characterized by elevated nucleotide diversity in multiple species. Microbial populations displayed varying degrees of homologous recombination and recombinant variants were often detected at 7–36% of loci genome-wide. Within multiple populations we identified genes with unusually high spatial differentiation of alleles, fewer recombinant events, elevated ratios of nonsynonymous to synonymous variants, and lower nucleotide diversity, suggesting recent selective sweeps for gene variants. Taken together, these results indicate that recombination and gene-specific selection commonly shape genetic variation in several understudied soil bacterial lineages.

## Introduction

Soil microbial communities play key biogeochemical roles in terrestrial ecosystems [1]. Within a single hectare of temperate grassland soil, there can be over 1000 kg of microbial biomass and corresponding large microbial population sizes [2]. Recent progress has been made in cataloging the diversity of 16S rRNA genes in soils [3], which is useful for understanding microbial community composition, but this technique is incapable of discerning most genetic variation within populations [4]. In addition, many of the most common soil microorganisms, such as the highly abundant *Acidobacteria*, *Verrucomicrobia*, and *Gemmatimonadetes* phyla, are underrepresented in or nearly absent from culture collections and genomic databases, even at the level of class or phylum [1, 5, 6]. For these reasons, the processes of recombination and selection in many of the most globally prolific soil microbial phyla remain unstudied. However, genome-resolved metagenomics, in which shotgun sequencing of metagenomic DNA is assembled and binned into draft genomes, has recently resulted in whole genome characterization of these rarely cultivated but widespread soil bacteria [7–11]. Sequence variation within genome-resolved metagenomic datasets can therefore be used to track changes in allele frequencies, and to infer the operation of evolutionary forces of genetic drift, natural selection, and homologous recombination in these natural populations [12, 13].

Homologous recombination can vary dramatically in its importance relative to the other processes for different microbial species. Analyses of reference genomes have shown that homologous recombination frequently occurs in bacteria populations, both globally and locally [14–16]. For example, certain populations of hotspring *Cyanobacteria* approach panmixia, where recombination is so frequent that individual cells are unlinked random mixtures of alleles [17]. In other species-like oceanic *Vibrio*, recombination is high but large blocks of alleles important for ecological niche differentiation are co-inherited and may remain linked due to selection [18, 19]. In soils, *Streptomyces flavogriseus* isolates were also found to approach a freely recombining panmixia [20]. In contrast, *Myxoccocus xanthus* isolates recovered from a series of soil samples were distinct but highly clonal, implying recombination between strains was low [21]. However, despite their frequent cultivation, cultivation-independent studies show that those taxa are comparatively rare in soils compared with undercultivated members of the *Acidobacteria*, *Gemmatimonadetes*, and *Verrucomicrobia* [1]. The degree of recombination in these rarely cultivated but abundant soil bacterial lineages has not been investigated, but recombination may be widespread, as high cell densities could promote the sharing of genetic material via transformation [22], conjugation [23], or the uptake of extracellular vesicles [24].

When recombination rates are low or selection is extremely strong, several clonal strains compete until one or more beneficial alleles is highly selected for, resulting in a single clonal genotype increasing in abundance or even sweeping to fixation. However, when recombination unlinks gene variants within a population, beneficial alleles can sweep through a population independent of genomic context in a selective sweep [25] with gene/locus-specific effects. One genome-resolved metagenomic study observed a single clonal sweep over a 9 year period for one *Chlorobium* population in a freshwater lake [26], while most of the other bacterial populations studied possessed genomic loci with unusually few SNPs (single nucleotide polymorphisms), an observation interpreted as evidence for gene-specific selective sweeps. However, positive selection acting on a genomic locus in a recombining population can also leave additional locus-specific signals, including higher linkage disequilibrium (a strong association between alleles) and different allele frequencies between populations [27, 28]. In soil microbial populations, comparative frequencies of gene-specific sweeps versus genome-wide clonal strain competition and replacement are largely unknown, as are the spatial scales at which these evolutionary processes can occur.

Previously, we conducted a large scale genome-resolved metagenomics study of soils from a grassland meadow in the Angelo Coast Range Reserve in northern California that established a dataset of 896 phylogenetically diverse microbial genomes dereplicated by species, and reported on community composition [29]. The soil at the site is a sandy loam mixture of ~45% clay-, ~45% silt-, and 10% sand-sized particles [8], with pH values in the range of 4.6–4.9. The mineralogy of the soils at the site was previously found to be predominantly vermiculite, with plagioclase and alkali feldspars and minor apatite [30]. The soils were classified as Ultic Haploxeralfs of the Holohan-Hollowtree-Casabonne complex, and at a depth of 30 cm were found to have a bulk density of ~2.0 g/cm^3^, a cation exchange capacity between 17 and 19 meq per 100 g soil, C:N ratios from 10–12, and total C concentrations of 10 mg g^−1^ up to 18 mg g^−1^ [30]. The grassland is dominated by annual Mediterranean grasses and forbs [8, 31]. The meadow has been part of a rainfall amendment climate change study ongoing for over 17 years and has also been studied in the context of plant diversity and productivity [32], invertebrate herbivores and predators [31], fungal communities [33], soil organic matter [30], metabolomics, and metaproteomics [8]. By analyzing the population genomics of 19 highly abundant bacterial species across this meadow, we found high nucleotide diversity within samples, intrapopulation genetic structure often shifting over local spatial scales, varying degrees of homologous recombination for different species, and gene-specific population differentiation partially driven by selection.

## Materials and methods

### Sampling, genome sequencing, and metagenomic assembly

**Fig. 1:**
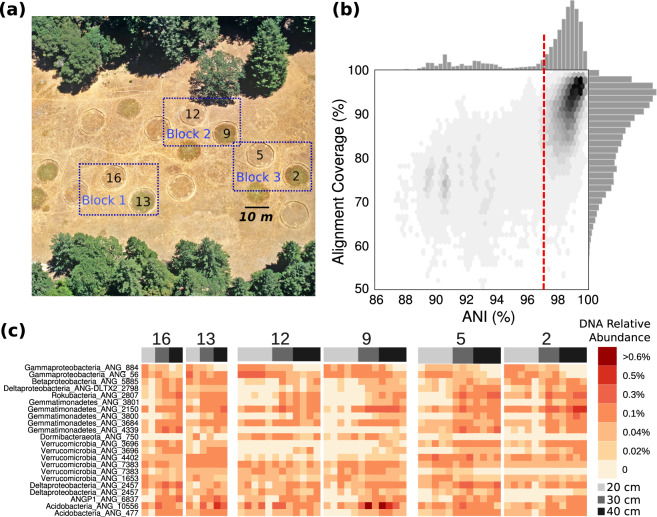
Meadow overview and population DNA relative abundances. **a** A bird’s eye view of the grassland meadow located at the Angelo Coast Range Reserve in Mendocino County, California (39° 44′ 17.7″ N, 123° 37′ 48.4″ W). **b** Histograms of average nucleotide identity (ANI) and alignment coverage (an approximation of % shared gene content) values for all-vs-all comparisons between genomes assembled from the meadow soils. **c** Relative DNA abundances of the 19 bacterial populations analyzed in this study in each sample, organized by experimental plot where sample was collected, ordered by sample depth from left to right.

The sampling scheme, local soil characteristics, and study design that were utilized in this analysis have been previously described [29]. Previously, 60 soil samples were collected at depths of 10–20, 20–30, and 30–40 cm near the respective centers of six 10 m diameter plots, spaced 5 m apart. Sampling plots were spatially arranged in “blocks” of two plots across the meadow, and one of the two plots in each block received extended spring rainfall [31]. Samples were collected over a period of 2 months before and following autumn rainfall, resulting in 10 samples per plot (Fig. 1a). Briefly, DNA was extracted from 10 g of soil for each sample using the PowerMax Soil DNA isolation kit (MoBio Laboratories) from individual soil cores. Metagenomic libraries were prepared and sequenced with 2 × 250 bp paired read sequencing on the Illumina HiSeq2500 platform at the Joint Genome Institute. Reads were quality filtered to a maximimum 200 bp in length using BBduk [34]. Metagenomes were assembled using IDBA_UD [35] and individual genomes were binned using differential coverage binning and a suite of metagenomic binners as previously described [29].

### Genome dereplication, filtering, and comparison

The 10,538 genome bins previously described obtained from the study site were dereplicated using dRep [36] with the secondary clustering threshold -sa 0.97, and were filtered with CheckM [37] to generate a dereplicated species-level (97% ANI) genome set to be used for read mapping with >70% completeness and <10% contamination. Representative genomes for each species cluster were chosen based on the highest CheckM completeness and lowest contamination using the scoring algorithm described by [36]. For this analysis, we then used the 19 species-level genome clusters with at least 12 replicate genomes that were estimated to be at least >80% complete with <10% contamination, independently assembled and binned out of different samples. Each of the 19 representative genomes was assembled from >100,000 short reads, and millions of reads could be assigned to each population from all 60 samples. Because microbial populations within the 10 g soil samples used for DNA extraction are comprised of orders of magnitude more cells than were sequenced, most read pairs are likely from unique cells (or DNA molecules) in the population. Each genome assembled from each sample was sequenced at around 10× coverage, but meadow-wide coverages for each population ranged from 224× to 908×(Supplementary Table S2**)**.

Open reading frames were predicted using Prodigal [38] and annotated using [1] USEARCH against UniProt [39], Uniref90 [40], and KEGG [41], and [2] HMM-based annotation of proteins using PFAM [42], and antiSMASH 4.0 [43] for biosynthetic gene prediction. PERMANOVA tests for association of ANI matrices with environmental data were run using the adonis2 function with the parameter ‘by’ set to ‘margin’ in the vegan package in R [44]. Multidimensional scaling (MDS) plots were made with the mds function from the smacof package in R, with the ndim parameter set to 4. Hypergeometric tests for statistical enrichment of protein families was performed using HMMER annotated PFAM features in R.

Possible contamination was further removed from representative genomes using the assembled replicate genomes for each species population. The pan-genomic analysis pipeline Roary [45] was run with default settings on the set of genomes for each species to identify protein clusters across genomes. Contigs with at least 50% of their protein clusters found in less than 25% of each genome set were then discarded as potential contamination (generally fewer than 20 contigs, often small, were removed per genome). Therefore, the final set of contigs used in this analysis contained only contigs that reliably assembled and binned independently for a species in multiple samples. Organism DNA relative abundances were calculated using the total number of reads that mapped to each genome in each sample and dividing by the total number of reads per sample.

### Read mapping, SNP calling, and nucleotide diversity

All metagenomic reads were mapped using Bowtie 2 [46] with default parameters except for the insert size parameter -X 1000 to an indexed database of all of the 664 dereplicated genomes obtained from the environment, a database which also contains representative genomes from each of our 19 species for study. Reads that mapped uniquely to the representative species of interest were then used for analysis. Read filtering of the resulting BAM files was performed using a custom script, filter_reads.py, available at the link under code availability. Mapping files were then filtered for reads that meet the following criteria: (1) both reads in a pair map within 1500 bp of each other to the same scaffold (as a maximum possible end to end insert size), (2) the combined read pair maps with a percent identity of at least 96% to the reference [3], at least one of the read pairs has a mapq score >1, indicating that this is a uniquely best mapping for this read pair in the index. We further compared nucleotide diversity of genes calculated with a 96% ANI_r2_ cutoff to those at a 98% ANI_r2_ cutoff and found a strong correlation (Fig. S1). SNPs were then called at frequencies >5% in each population using reads from all samples, using a simple null model that assumes a false discovery rate <~10^−6^.

For all population diversity metrics, we used reproducible custom python scripts (available under Code Availability) that calculated metrics, each explained below, from all filtered cross-sample read mappings. For each representative genome in our set of 19, we analyzed its data in samples that passed a cutoff of at least 50% of the genome being covered with at least 5× coverage. Five hundred eighty-six out of 1140 sample genome comparisons (19 genomes × 60 samples) passed this minimum requirement. Base pairs of reads with Phred scores less than 30 were not used in SNP or linkage analyses. Nucleotide diversity was calculated as the expected frequency of a difference between two sequencing reads at a position, equation pi=1 − (*A*^2^ + *C*^2^ + *G*^2^ + *T*^2^), where *A*, *C*, *G*, *T* and the observed proportions of each respective nucleotide. This is equivalent to the definition from [47] calculated separately on each genomic position with at least 5× coverage within each sample, and then averaged across genes. Sample read mappings were pooled by replicates, by plot of origin, by block and origin, and finally by all samples in the meadow, and nucleotide diversity was recalculated on each pooled set of samples. For downstream analyses, nucleotide diversity for all samples (meadow-wide, Fig. 3) and nucleotide diversity within each of the three sampling blocks (block-wide, Fig. 5) was analyzed. To quantify the impact of changing sequencing coverage on nucleotide diversity, we recalculated nucleotide diversity for each genome, subsampling coverage at each genomic position (Fig. S2). We found that the bias in nucleotide diversity due to low sequencing coverage was minimal above 50×, and our block-wide and meadow-wide coverages are often well above this coverage. We see a larger bias in nucleotide diversity when subsampling to only 5x coverage, but this bias is small compared with the biological variation observed between samples, and many of our individual soil samples are over 10×.

SNPs were called on the combined meadow-wide population set of filtered reads. We constructed a simple null model based on an error rate of 0.01% (the Phred 30 cutoff) and simulated simple sampling with replacement to construct estimated rates of erroneous SNP counts at genomic positions of varying coverages. Positions with an alternative allele that occurred with counts that had a false positive rate of ~10^−6^ with the given coverage of that site in the null model and a minimum allele frequency (MAF) of 5% were called as SNPs. Because meadow-wide coverages were at least 224× (and ranged up to 908×) the MAF cutoff alone would likely be a stringent cutoff for Phred 30 error rates. SNPs were assigned as synonymous or nonsynonymous using a custom BioPython script and the gene calls annotated by Prodigal.

### Linkage disequilibrium, FST, and tests for selection

Linkage was calculated within mapped reads for all pairs of segregating sites that were spanned by at least 30 read pairs with high quality base pair data. *R*^2^ and *D*′ linkages were calculated using formulas described by [48]. The relative rate of recombination to mutation (gamma/mu) for each population was calculated using the mcorr package (cite) on synonymous third position codon sites across filtered mapped reads from all samples. We analyzed 10 out of 19 genomes, as these had normally distributed residuals for the model fit and the bootstrapping mean was within 2× of the final estimate for gamma/mu. *F*_ST_, (a measure of differences in allele frequencies between two populations), was calculated on sites segregating across both blocks being compared (for all three block comparisons) using the Hudson method [49] as recommended by [50], as implemented in the scikit-allel package [51]. A site had to have a coverage of at least 20× in each block in order to calculate *F*_ST_, and genes which had coverages in a block outside of the range of two standard deviations were excluded from the analysis. A ratio of averages was then used to determine mean *F*_ST_ for each gene. A two-sample Wilcoxon test was used to determine if average linkage of highly differentiated loci differed from the genomic average for each species, and two-sample *t*-tests in R [44] were used to determine if average nucleotide diversity of highly differentiated loci differed from the genomic average. Both sets of tests were corrected for multiple hypotheses using the Benjamini–Hochberg [52] method.

## Results

### Genomic similarity within bacterial populations is spatially organized across a meadow

Starting with an undereplicated but quality filtered dataset of 3215 draft quality genomes assembled from samples collected from across the meadow [29], we calculated all pairwise genome-wide average nucleotide identities (ANI) and alignment coverages (roughly analogous to shared gene content; Fig. 1b). We observed a sharp decrease in pairwise ANI for all of the genomes from the meadow around 96.5–97%, similar to the threshold for bacterial species delineation reported recently [53]. There was a more gradual decline in shared gene content. We used the 97% ANI cutoff to cluster genomes into groups of species-like populations and found that some species-like groups contained dozens of near-complete draft genomes, each assembled from a different sample independently. To focus on the populations that were most abundant in the metagenomic data, we selected species clusters with at least 12 genomes estimated to be >80% complete with <10% contamination for population genetics analysis, which resulted in a final set of 467 genomes from 19 widespread species populations (312 genomes are estimated to be >90% complete; Supplementary Table S1). The bacterial species in this set included many commonly reported highly abundant soil bacteria from phyla including *Chloroflexi*, *Acidobacteria*, *Verrucomicrobia*, and *Candidatus* Rokubacteria, which are known to be abundant globally in soils [1, 6], but remain understudied. Most of the species in this set were likely novel at the taxonomic rank of class, and one likely represents a novel candidate phylum tentatively designated *Candidatus* ANGP1 (Diamond et al. 2019). Based on measurement of the relative DNA abundance of each population across the entire meadow, these bacteria are some of the most abundant species in the soil, although no individual species contributed >1% of the DNA in a sample (Fig. 1c).

**Fig. 2:**
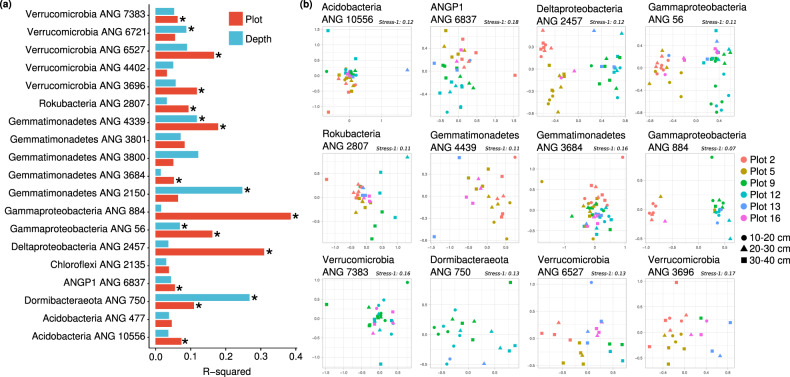
Spatial variation in genetic differences within species. **a** The percentage of variation in genetic similarity (ANI) of consensus genomes explained by plot of origin (red) and sampling depth (blue). **b** Multidimensional scaling ordinations of genetic dissimilarities between genomes within each species, for all 12 populations for which sampling plot explained a significant fraction of the variation in genetic dissimilarity (PERMANOVA; FDR = 5%; *p* < 0.05). The first two axes are plotted, there is a single point for each genome independently assembled for a population, and genomes are colored by sample plot of origin and the point shape indicates the sample depth.

For each of the 19 meadow-wide populations, we tested to see if the meadow plot of origin predicted genetic similarity of the assembled genomes (PERMANOVA; FDR ≤5 %; adjusted *p* ≤ 0.05). Further, we tested if genomes obtained from the same soil depth were more similar than those collected from different depths. We found that the genetic variation of genomes from 12 of the 19 populations were significantly associated with sampling plot, and that genetic variation within 5 of the 19 populations were significantly associated with sampling depth (Fig. 2a). MDS of the nucleotide identity matrices of genomes from each population shows clear associations with both plot of origin and depth (Fig. 2b). Because the genome assembly from each sample reflects the most abundant sequence variant in each population, this implies that major allele frequencies varied across the meadow for a majority of the populations. While local spatial heterogeneity has been shown to highly explain microbial community composition in soils [54], here we demonstrate that there are also spatial patterns within the genetic variation of some individual species.

### Population nucleotide diversity is high meadow-wide and within soil samples

**Fig. 3:**
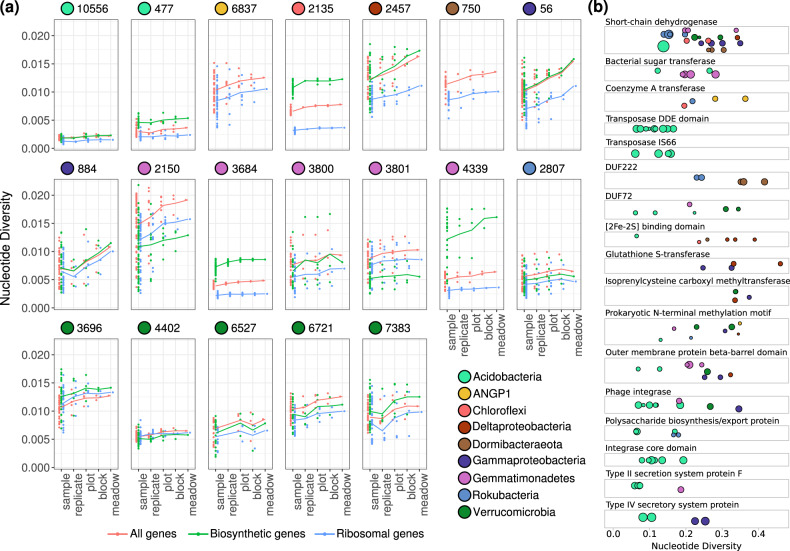
Nucleotide diversity of 19 highly abundant bacterial populations. **a** Distributions of average per-gene nucleotide diversity for each population, measured across increasing scales of sampling. Separated by all genes (red), ribosomal genes (blue), and biosynthetic genes (green) for species that have them (all except species *Candidatus* ANGP1 6837 and *Dormibacteraeota* ANG 750). Lines connect the means of each distribution of points across scales. **b** Nucleotide diversities of genes from protein families that were found to be enriched among genetically diverse genes compared with the average genomic frequencies. Each gene is a point arranged by protein family from the PFAM database, and point size scales with the gene’s length.

To assess the genetic variability within each population across the meadow, we calculated the per-site nucleotide diversity of the sequencing reads at each locus for that population. Metagenomic studies have sometimes used either the average similarity of reads to a reference or the total number of SNPs/Mbp as metrics of genetic diversity [26, 55]. We chose to measure nucleotide diversity, also previously measured by [56], because [1] it is less sensitive to large changes in coverage (Figs. S1 and S2) [2], it can be calculated both for a single site and averaged over genes or windows, and [3] it considers not only the number of SNPs but also their frequencies in the population. We found a wide range of per-gene nucleotide diversity values for the 19 different populations (Fig. 3a). Because nucleotide diversity is less sensitive to changes in coverage, we could use it to track how nucleotide diversity changes between the plots spread across the meadow. We calculated nucleotide diversity in pooled mapped reads for each population sampled from the same location and soil depth, within plot, within block (pairs of plots), and across the entire meadow for each species (Fig. 3a). Although nucleotide diversity tends to be higher at the meadow scale compared with the sample scale, the nucleotide diversity within some samples was comparable to that across the entire meadow for many populations, indicating that in some cases high nucleotide diversity persists within soils even at the centimeter scale.

In almost all populations, the ribosomal genes consistently had lower nucleotide diversity than the average genes in the genome, consistent with these genes being strongly conserved and under higher purifying selection [57] (Fig. 3a). Biosynthetic genes involved in the production of small molecules, annotated with *antiSMASH* [43], were found to have significantly higher nucleotide diversity than the genomic average in *Chloroflexi* and one *Acidobacteria* species, while having significantly lower nucleotide diversity in one *Gemmatimonadetes* species (Welch two-sample *t* test; *q* < 0.05)(Fig. 3a). Examining all genetically diverse genes with nucleotide diversities greater than 2.5 standard deviations above the mean within each population, we find that protein families for biosynthesis of small molecules and extracellular secretion were significantly over-enriched compared with the genomic averages (hypergeometric test; *p* < 0.05) (Fig. 3b). Short chain dehydrogenase enzymes and outer membrane beta-barrel domains were significantly enriched among highly diverse genes across several taxa, whereas multiple transposon families were diversifying within *Acidobacteria* genomes. Across the *Acidobacteria*, *Gammaproteobacteria*, and *Gemmatimonadetes* species studied, secretion system proteins were also diversifying. These genes involved in biosynthesis and secretion may likely have experienced local selective pressures for diversification across soil microbial species.

### Homologous recombination is common, but populations exist far from panmictic equilibria

**Fig. 4:**
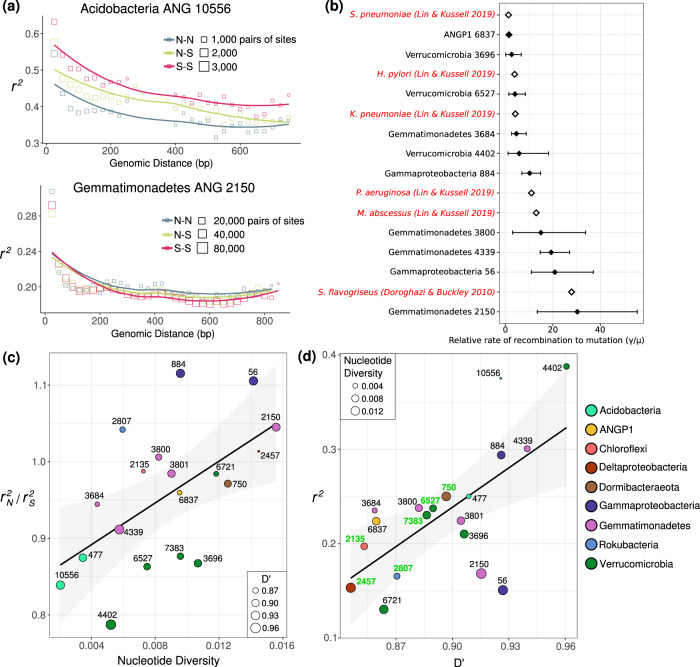
Varying rates of linkage disequilibrium within populations. **a** Linkage decay of *r*^2^ for pairs of loci within the population with the lowest nucleotide diversity (top) and the highest nucleotide diversity (bottom). Each square is an average of pairs of biallelic sites at that distance, with the area of the square point proportional to the number of pairs of biallelic sites that went into the mean. Haplotypes (site pairs) are binned by the predicted function of the mutations of each of the paired SNPs (nonsynonymous: N, synonymous: S). **b** Relative rates of recombination to mutation calculated across the entire meadow for ten populations on synonymous third position codon sites, compared with previous values (red) reported by Lin and Kussell [16] and the value reported for Streptomyces flavogensis by Doroghazi and Buckley [20]. Error bars represent the 95% confidence interval across 1000 bootstraps. **c** The relationship between nucleotide diversity and *r*^*2*^_N_/*r*^*2*^_S_. The mean nucleotide diversity and the mean ratio of the linkage of nonsynonymous-nonsynonymous vs synonymous-synoymous pairs of mutations across species is shown. The size of each point represents the mean *D*′ value for that species. A linear regression is shown (linear regression; *R*^2^ = 0.29; *p* = 0.009). **d** The relationship between mean *r*^2^ and mean *D*′ across the 19 bacterial populations studied. Genomes with evidence for multiple operonic competence related genes are labeled in green. A linear regression model is shown (*F*-statistic: 11.9, Adjusted *R*-squared: 0.38, *p* = 0.003).

Measuring the impact of homologous recombination on the observed genetic diversity in a population can be accomplished with metagenomic data by measuring linkage disequilibrium of SNPs spanned by paired reads [16, 17]. When recombination occurs within a population, the chance for a recombination event to occur between two sites on the genome increases with the distance between them, resulting in a characteristic signal known as linkage decay. Given ~200 bp reads and intra-read pair distances with a median of 383 bp and a 95th percentile of 500 bp, we could reliably assess genomic linkage of SNPs from 772 bp (median) to 846 bp (95th percentile) bp apart in each population. Consistent with the expectation that natural bacterial populations can undergo extensive homologous recombination, we observed the *r*^2^ metric of linkage disequilibrium decay as the genomic distance between two polymorphisms increased (Fig. 4a; Fig. S3). Using the *mcorr* package, we could estimate the neutral rate of recombination relative to mutation on synonymous third position codon sites for 10 out of 19 populations. While the estimated confidence intervals for these relative rates were large and varied between species, they were well within the ranges reported in the literature [16] for many known highly recombinogenic species, but generally were below the rate reported for a *S. flavogriseus* population considered to be approaching panmixia [20].

In the less genetically diverse populations, there was a noticeable higher *r*^2^ of synonymous variants linked to other synonymous variants than for nonsynonymous variants linked to other nonsynonymous variants (Fig. 4c). This has been previously observed in hotspring *Cyanobacteria*, and was explained as a decrease in coupling linkage for slightly deleterious nonsynonymous variants, where recombinants inheriting the doubly deleterious haplotype (of a pair of variants) are selected against [58]. In more genetically diverse populations, this ratio shifts toward nonsynonymous polymorphisms (*r*^*2*^_N_) having higher *r*^*2*^ than synonymous (*r*^*2*^_S_) (Fig. 4c). One recent study reported a similar positive *r*_N_-*r*_S_ ratio as a signature of positive balancing selection in *Neisseria gonorrhoeae* [59]; for six of the 19 populations analyzed here, the genomic average *r*^*2*^_N_/*r*^*2*^_S_ ratio was greater than 1 (Fig. 4c), also indicating a greater degree of coupling linkage for nonsynonymous variants in these populations. The increase in the *r*^*2*^_N_/*r*^*2*^_S_ ratio with nucleotide diversity (linear regression; *R*^2^ = 0.29; *p* = 0.009) suggests an increase in the ratio of beneficial to slightly deleterious nonsynonymous SNPs as diversity increases.

Although *r*^2^ is often used as a signal to identify the presence or absence of recombination, *r*^2^ values <1 can occur with or without recombination. For example, three of four possible haplotypes (pairs of variants) for two biallelic sites could occur due to lineage divergence prior to mutation occurring at one of the sites. *D*′, an alternative metric of linkage equilibrium, is only <1 if all possible combinations of a pair of biallelic sites are observed [48], which can only occur in the presence of recombination or recurrent mutation. Generally, we found that average *D*′ for a population linearly correlated with mean *r*^2^ (Fig. 4d). Inspection of the distribution of pairs of SNPs separated by <~1 kb revealed that 3 of 4 possible haplotypes is most common, but there was detection of all four possible biallelic haplotype combinations (*D*′ < 1), at 7% to 36% of all site pairs in each population (Fig. S4). The observed frequencies of the least common of the four SNP combinations also were higher than expected based on sequencing error, although much less frequent than expected from linkage equilibrium, as evidenced by mean *D*′ values above 0.8. Nonetheless, the extensive appearance of *D*′ < 1 at a significant fraction of loci, along with a signal of linkage decay with genomic distance across all populations, provides firm evidence for ongoing processes of within population homologous recombination, albeit to different degrees between organisms.

Given evidence for recent homologous recombination, we searched the genomes for genes that could confer natural competence, such as homologs of the *ComEC* gene with all three functional domains [60], and identified loci with additional genes involved in DNA uptake and recombination [61] (Supplementary Table S3). We found that the presence of a ComEC homolog with multiple adjacent operonic recombination-related genes was strongly associated with the lowest values of D’ (Fig. 4d). Thus, it is likely that natural competence is a common mechanism that facilitates homologous recombination for abundant soil bacteria.

### Gene-specific selective sweeps contribute to divergence of alleles across the meadow

In neutrally evolving local populations, nucleotide diversity is expected to increase monotonically with population size [47]. Across populations, as we did not see a relationship between nucleotide diversity and relative abundance (Fig. S5; linear regression; *p* = 0.88), purely neutral growth and processes cannot explain the observed differences in nucleotide diversity between these populations. Similarly, we also did not observe a significant relationship between diversity and abundance within species for 14 of the 19 populations (Fig. S5). Except for populations with the lowest nucleotide diversity, ratios of nonsynonymous to synonymous polymorphisms for each population are consistently low. This trend, as also observed in lake metagenomics [62] and whole genome comparisons [63], further indicates that purifying selection has eliminated slightly deleterious mutations in the populations that have accrued more nucleotide diversity (Fig. S6; linear regression; R^2^ = 0.25; p = 0.018). In all species except the least diverse population (an *Acidobacterium*), nonsynonymous variants also had consistently higher values of *D*′ than synonymous variants. Further, as genome-wide D’ decreased (more recombination), the degree to which nonsynonymous variants were more linked than synonymous (D’_N_/D’_S_) increased (Figs S7; S8). As the number of observed recombination events increased, nonsynonymous linkage increased in comparison to synonymous linkage. This effect is consistent with stronger selection on nonsynonymous variants with an increase in diversity: both purifying selection and positive selection would increase D’ for nonsynonymous SNPs.

Soil ecosystems are exceptionally heterogeneous, and environmental factors can change over millimeter distances, potentially due to changes in aboveground plant productivity, soil geochemistry, plant litter composition, and soil particulate structure [64]. While it is difficult to tease apart the effects of changing abiotic parameters over spatial scales, it is possible to examine how allele frequencies change over the scales within our study design. We calculated the pairwise fixation index *F*_*ST*_ for each gene between allele frequencies from the three meadow blocks for every species-group (Fig. 1b). For most populations, mean gene *F*_*ST*_ values were low (<5%), consistent with dispersal of most alleles between blocks (Fig. S9). For a minority of populations, mean gene *F*_*ST*_ values were consistently >10%, indicating that there was significant geographic organization of genetic structure at most loci across the genome (Supplementary Table S4). Therefore, while the total variation in genome-wide major consensus alleles is often well explained by meadow geography, most individual alleles have a high chance of being found at fairly similar frequencies across the meadow.

**Fig. 5:**
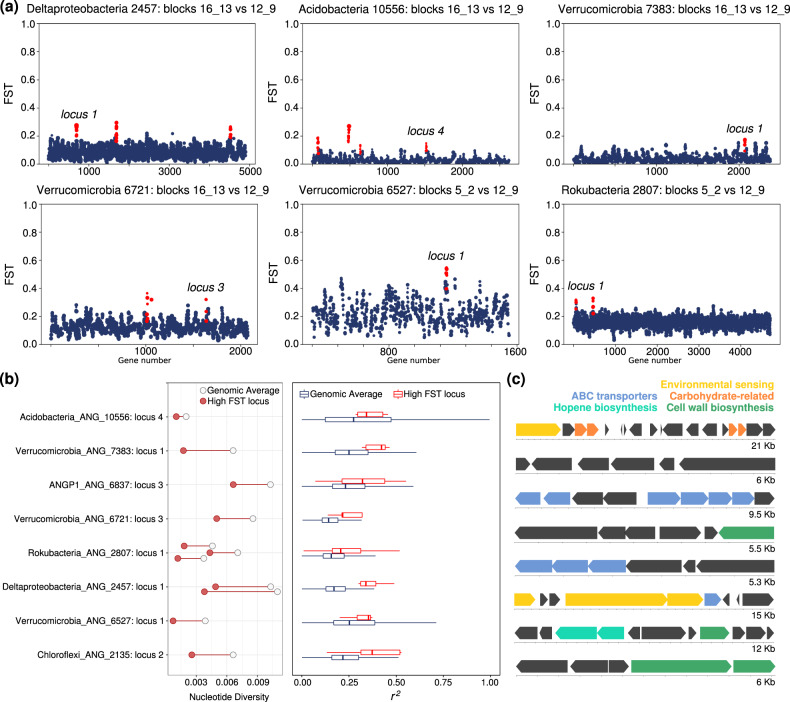
Highly differentiated genomic loci between sites within a meadow. **a** Values of *F*_ST_ for genes across the genomes of six bacterial populations. Each point is a gene, and the size of the point is determined by the number of SNPs within that gene. Plotted is the mean *F*_ST_ for that gene. Loci with significantly higher *F*_ST_ than the background are highlighted in red, and those that passed further filtering are labeled by their genome-specific locus numbers used in part (**b**). **b** Left: Nucleotide diversity at highly differentiated loci (red circle) compared with the average (empty circle) for each population. Right: The extent of linkage disequilibrium at highly differentiated loci (red) compared with the genomic average (black) for each population. **c** Gene diagrams and annotations of highly differentiated loci (genome and loci identities given in **b**). Each block indicates an open reading frame, and blocks are colored by a subset of predicted functions.

When specific loci are characterized by significantly higher *F*_*ST*_ than the background average for the genome, it is characteristic of population-specific (in this case, spatially defined) selective pressures acting on that locus [65]. To identify genomic regions of unexpectedly high *F*_*ST*_, we scanned over a moving 5 gene window and tested if that region had a mean *F*_*ST*_ greater than 2.5 standard deviations above the genomic mean. We removed genes with either coverage 2 standard deviations above or below the mean coverage in either block from this analysis (Supplementary Table S5). To define the length of the genomic region with elevated *F*_*ST*_ values, we extended successful windows until the mean *F*_*ST*_ fell below this cutoff. This test at first identified 48 loci of elevated *F*_*ST*_ within some microbial genomes, despite those genomes having low average *F*_*ST*_ (Fig. S10). To further test for evidence of recent selection at these loci we looked for a statistically significant average increase in linkage and a significant change in nucleotide diversity compared with the genomic average in one or both of the blocks (Fig. 5a). We noticed that both signals of purifying selection (characterized by low N:S ratios) and a reduction in genomic coverage (potentially indicating gene loss in some portion of the population) often correlated with low nucleotide diversity in genomic regions, and based on this, caution against identifying gene-specific selective sweeps in metagenomic data based solely on a reduction in nucleotide diversity or SNP frequency. While we found many loci with unusual *F*_*ST*_ or strong changes in nucleotide diversity, our stringent criteria narrowed that set down to 8 high *F*_*ST*_ loci with significantly increased rates of linkage compared with the genomic background (Fig. 5c**;** Fig. S10). These loci also had significant changes in nucleotide diversity within blocks when compared with their genomic averages (Fig. 5b, c). All of these loci showed decreases in nucleotide diversity, consistent with selective sweep events in one or multiple meadow blocks. Genes at these loci also had higher N:S ratios than genomic averages, possibly consistent with either recent selection acting on beneficial nonsynonymous mutations or a local accumulation of slightly deleterious nonsynonymous genetic hitchhikers.

Some loci with evidence of recent differential selection across the meadow contained transporter genes (Fig. 5c, Supplementary Table S6), which could indicate selective pressures for uptake of different compounds between sites. A *Verrucomicrobia* population also showed evidence of a selective sweep occurring at a putative hopene biosynthesis operon, often involved in regulating membrane stability. Within a *Deltaproteobacteria* population, a highly differentiated locus encoded for numerous genes involved in two-component systems and histidine kinases, potentially related to environmental sensing and response. Taken together, these multiple genomic signals suggest that gene-specific selection partially drives differences in population genetic structure across meadow soils.

## Discussion

Developing a cohesive picture of the genetic structures of soil bacterial populations is crucial for understanding the evolution and distribution of genes that can play critical ecosystem and niche-specific functions. Doing so has been difficult because the most abundant soil bacteria are difficult to cultivate, and when cultivation is possible, it is uncertain if microbial isolates are truly random samples of a population, considering the intense selective pressure put on cells during the isolation process. Here, we show that recent advances in sequencing technologies and software tools enable the monitoring of heterogeneity within genomically defined bacterial populations needed to address these questions in soil. Compared with a previously published study on freshwater lake microbial populations [26], we observed far more polymorphisms per species (4721 to 43,225 SNPs/Mb, Supplementary Table S2), despite having a MAF cutoff of 5%. Most populations also had higher rates of SNPs/Mb than a similar analysis of metagenome assembled genomes found for microbial populations in deep-sea hydrothermal vents [55]. Nucleotide diversity was heterogeneous within individual samples but was often still high, implying that within each 10 g sample of soil many alternative alleles are frequently encountered.

The results of this study suggest that recombination and gene-specific selection are important modes of evolution across the most abundant soil microbes, and are capable of structuring populations at a scale of meters in soils. Even within a single meadow, our data demonstrate that there are likely thousands of combinatorial genetic mixtures for each species, with recombination resulting in no easily measurable numbers of irreducible strain-like lineages. We were able to calculate the relative rate of recombination to mutation for half of our species studied, and comparing these values to those reported for other species in the literature places them among other known recombinogenic species. Thus, the dynamics of dominant soil bacterial populations may be partially described using ‘quasi-sexual’ models [12, 17]. Although we only observed the importance of these dynamics in structuring populations at the 1–10 m scale, it is still uncertain how evolutionary dynamics in soil bacterial populations develop on other spatial and temporal scales. We conclude that future work on soil microbial ecology would benefit by considering the role of substantial unlinked allelic diversity within species in shaping local gene content and allele frequencies.

## Supplementary information

Supplementary Figure Legends

Supplementary Figure S1

Supplementary Figure S2

Supplementary Figure S3

Supplementary Figure S4

Supplementary Figure S5

Supplementary Figure S6

Supplementary Figure S7

Supplementary Figure S8

Supplementary Figure S9

Supplementary Figure S10

Supplementary Tables S1-S6

## Data Availability

The published genomes and raw sequencing reads for this study are available under NCBI BioProject number PRJNA449266. Large data tables and genome sequences are available at: https://figshare.com/projects/Soil_bacterial_populations_are_shaped_by_recombination_and_gene-specific_selection_across_a_meadow/65789.

## Code availability

Reproducible notebooks, analyses and additional data tables used in this analysis are available at https://github.com/alexcritschristoph/soil_popgen/.

## References

[1] Fierer N (2017). Embracing the unknown: disentangling the complexities of the soil microbiome. Nat Rev Microbiol.

[2] Fierer N, Strickland MS, Liptzin D, Bradford MA, Cleveland CC (2009). Global patterns in belowground communities. Ecol Lett.

[3] Thompson LR, Sanders JG, McDonald D, Amir A, Ladau J, Locey KJ (2017). A communal catalogue reveals Earth’s multiscale microbial diversity. Nature.

[4] Chevrette MG, Carlos-Shanley C, Louie KB, Bowen BP, Northen TR, Currie CR (2019). Taxonomic and metabolic incongruence in the ancient genus Streptomyces. Front Microbiol.

[5] Lloyd KG, Steen AD, Ladau J, Yin J, Crosby L (2018). Phylogenetically novel uncultured microbial cells dominate earth microbiomes. mSystems.

[6] Bergmann GT, Bates ST, Eilers KG, Lauber CL, Caporaso JG, Walters WA (2011). The under-recognized dominance of Verrucomicrobia in soil bacterial communities. Soil Biol Biochem.

[7] Hultman J, Waldrop MP, Mackelprang R, David MM, McFarland J, Blazewicz SJ (2015). Multi-omics of permafrost, active layer and thermokarst bog soil microbiomes. Nature.

[8] Butterfield CN, Li Z, Andeer PF, Spaulding S, Thomas BC, Singh A (2016). Proteogenomic analyses indicate bacterial methylotrophy and archaeal heterotrophy are prevalent below the grass root zone. PeerJ.

[9] White RA, Bottos EM, Chowdhury TR, Zucker JD, Brislawn CJ, Nicora CD (2016). Moleculo long-read sequencing facilitates assembly and genomic binning from complex soil metagenomes. mSystems.

[10] Ji M, Greening C, Vanwonterghem I, Carere CR, Bay SK, Steen JA (2017). Atmospheric trace gases support primary production in Antarctic desert surface soil. Nature.

[11] Woodcroft BJ, Singleton CM, Boyd JA, Evans PN, Emerson JB, Zayed AAF (2018). Genome-centric view of carbon processing in thawing permafrost. Nature.

[12] Garud NR, Good BH, Hallatschek O, Pollard KS (2019). Evolutionary dynamics of bacteria in the gut microbiome within and across hosts. PLoS Biol.

[13] Whitaker RJ, Banfield JF (2006). Population genomics in natural microbial communities. Trends Ecol Evol.

[14] González-Torres P, Rodríguez-Mateos F, Antón J, Gabaldón T (2019). Impact of homologous recombination on the evolution of prokaryotic core genomes. MBio.

[15] Sakoparnig T, Field C, van Nimwegen E. Whole genome phylogenies reflect long-tailed distributions of recombination rates in many bacterial species. https://www.biorxiv.org/content/10.1101/601914v1. 2019. 10.7554/eLife.65366 PMC7884076 33416498

[16] Lin M, Kussell E (2019). Inferring bacterial recombination rates from large-scale sequencing datasets. Nat Methods.

[17] Rosen MJ, Davison M, Bhaya D, Fisher DS (2015). Fine-scale diversity and extensive recombination in a quasisexual bacterial population occupying a broad niche. Science.

[18] Cui Y, Yang X, Didelot X, Guo C, Li D, Yan Y (2015). Epidemic clones, oceanic gene pools, and eco-LD in the free living marine pathogen Vibrio parahaemolyticus. Mol Biol Evol.

[19] Jesse Shapiro B, Friedman J, Cordero OX, Preheim SP, Timberlake SC, Szabó G (2012). Population genomics of early events in the ecological differentiation of bacteria. Science.

[20] Doroghazi JR, Buckley DH (2010). Widespread homologous recombination within and between Streptomyces species. ISME J.

[21] Wielgoss S, Didelot X, Chaudhuri RR, Liu X, Weedall GD, Velicer GJ (2016). A barrier to homologous recombination between sympatric strains of the cooperative soil bacterium *Myxococcus xanthus*. ISME J.

[22] Thomas CM, Nielsen KM (2005). Mechanisms of, and barriers to, horizontal gene transfer between bacteria. Nat Rev Microbiol.

[23] Rocha EPC, Cornet E, Michel B (2005). Comparative and evolutionary analysis of the bacterial homologous recombination systems. PLoS Genet.

[24] Tran F, Boedicker JQ (2019). Plasmid characteristics modulate the propensity of gene exchange in bacterial vesicles. J Bacteriol.

[25] Smith JM, Haigh J (2007). The hitch-hiking effect of a favourable gene. Genet Res.

[26] Bendall ML, Stevens SL, Chan L-K, Malfatti S, Schwientek P, Tremblay J (2016). Genome-wide selective sweeps and gene-specific sweeps in natural bacterial populations. ISME J.

[27] Krause DJ, Whitaker RJ (2015). Inferring speciation processes from patterns of natural variation in microbial genomes. Syst Biol.

[28] Shapiro BJ, David LA, Friedman J, Alm EJ (2009). Looking for Darwin’s footprints in the microbial world. Trends Microbiol.

[29] Diamond S, Andeer PF, Li Z, Crits-Christoph A, Burstein D, Anantharaman K, et al. Mediterranean grassland soil C–N compound turnover is dependent on rainfall and depth, and is mediated by genomically divergent microorganisms. Nature Microbiol. 2019. 10.1038/s41564-019-0449-y PMC6784897 31110364

[30] Berhe AA, Suttle KB, Burton SD, Banfield JF (2012). Contingency in the direction and mechanics of soil organic matter responses to increased rainfall. Plant Soil.

[31] Suttle KB, Thomsen MA, Power ME (2007). Species interactions reverse grassland responses to changing climate. Science.

[32] Sullivan MJP, Thomsen MA, Suttle KB (2016). Grassland responses to increased rainfall depend on the timescale of forcing. Glob Chang Biol.

[33] Hawkes CV, Kivlin SN, Rocca JD, Huguet V, Thomsen MA, Suttle KB (2011). Fungal community responses to precipitation. Glob Change Biol.

[34] Bushnell B. BBTools software package. http://sourceforge.net/projects/bbmap. 2014.

[35] Peng Y, Leung HC, Yiu SM, Chin FY (2012). IDBA-UD: a de novo assembler for single-cell and metagenomic sequencing data with highly uneven depth. Bioinformatics.

[36] Olm MR, Brown CT, Brooks B, Banfield JF (2017). dRep: a tool for fast and accurate genomic comparisons that enables improved genome recovery from metagenomes through de-replication. ISME J.

[37] Parks DH, Imelfort M, Skennerton CT, Hugenholtz P, Tyson GW (2015). CheckM: assessing the quality of microbial genomes recovered from isolates, single cells, and metagenomes. Genome Res.

[38] Hyatt D, Chen G-L, Locascio PF, Land ML, Larimer FW, Hauser LJ (2010). Prodigal: prokaryotic gene recognition and translation initiation site identification. BMC Bioinforma.

[39] UniProt Consortium T. (2018). UniProt: the universal protein knowledgebase. Nucleic Acids Res.

[40] Suzek BE, Wang Y, Huang H, McGarvey PB, Wu CH (2015). UniProt Consortium. UniRef clusters: a comprehensive and scalable alternative for improving sequence similarity searches. Bioinformatics.

[41] Kanehisa M, Goto S (2000). KEGG: kyoto encyclopedia of genes and genomes. Nucleic Acids Res.

[42] Finn RD, Bateman A, Clements J, Coggill P, Eberhardt RY, Eddy SR (2013). Pfam: the protein families database. Nucleic Acids Res.

[43] Blin K, Wolf T, Chevrette MG, Lu X, Schwalen CJ, Kautsar SA (2017). antiSMASH 4.0—improvements in chemistry prediction and gene cluster boundary identification. Nucleic Acids Res.

[44] R Development Core Team. The R reference manual: base package. Network Theory. 2003. 736 p.

[45] Page AJ, Cummins CA, Hunt M, Wong VK, Reuter S, Holden MTG (2015). Roary: rapid large-scale prokaryote pan genome analysis. Bioinformatics.

[46] Langmead B, Salzberg SL (2012). Fast gapped-read alignment with Bowtie 2. Nat Methods.

[47] Nei M, Li WH (1979). Mathematical model for studying genetic variation in terms of restriction endonucleases. Proc Natl Acad Sci USA.

[48] VanLiere JM, Rosenberg NA (2008). Mathematical properties of the r^2^ measure of linkage disequilibrium. Theor Popul Biol.

[49] Hudson RR, Slatkin M, Maddison WP (1992). Estimation of levels of gene flow from DNA sequence data. Genetics.

[50] Bhatia G, Patterson N, Sankararaman S, Price AL (2013). Estimating and interpreting FST: the impact of rare variants. Genome Res.

[51] Miles A, Harding N.cggh/scikit-allel: v1. 1.8. 10.5281/zenodo.822784. 2017

[52] Benjamini Y, Hochberg Y (1995). Controlling the false discovery rate: a practical and powerful approach to multiple testing. J R Stat Soc: Ser B (Methodol).

[53] Jain C, Rodriguez-R LM, Phillippy AM, Konstantinidis KT, Aluru S (2018). High throughput ANI analysis of 90K prokaryotic genomes reveals clear species boundaries. Nat Commun.

[54] O’Brien SL, Gibbons SM, Owens SM, Hampton-Marcell J, Johnston ER, Jastrow JD (2016). Spatial scale drives patterns in soil bacterial diversity. Environ Microbiol.

[55] Anderson RE, Reveillaud J, Reddington E, Delmont TO, Eren AM, McDermott JM (2017). Genomic variation in microbial populations inhabiting the marine subseafloor at deep-sea hydrothermal vents. Nat Commun.

[56] Jordan IK, Rogozin IB, Wolf YI, Koonin EV (2002). Essential genes are more evolutionarily conserved than are nonessential genes in bacteria. Genome Res.

[57] Rosen MJ, Davison M, Fisher DS, Bhaya D (2018). Probing the ecological and evolutionary history of a thermophilic cyanobacterial population via statistical properties of its microdiversity. PLoS ONE.

[58] Arnold BJ, Sohail M, Wadsworth C, Corander J, Hanage WP, Sunyaev S, et al. Fine-scale haplotype structure reveals strong signatures of positive selection in a recombining bacterial pathogen. https://www.biorxiv.org/content/10.1101/634147v1. 2019. 10.1093/molbev/msz225 PMC6993868 31589312

[59] Pimentel ZT, Zhang Y (2018). Evolution of the natural transformation protein, ComEC, in bacteria. Front Microbiol.

[60] Cassier-Chauvat C, Veaudor T, Chauvat F (2016). Comparative genomics of DNA recombination and repair in cyanobacteria: biotechnological implications. Front Microbiol.

[61] Shapiro BJ (2016). How clonal are bacteria over time?. Curr Opin Microbiol.

[62] Rocha EPC, Smith JM, Hurst LD, Holden MTG, Cooper JE, Smith NH (2006). Comparisons of dN/dS are time dependent for closely related bacterial genomes. J Theor Biol.

[63] Vos M, Wolf AB, Jennings SJ, Kowalchuk GA (2013). Micro-scale determinants of bacterial diversity in soil. FEMS Microbiol Rev.

[64] Holsinger KE, Weir BS (2009). Genetics in geographically structured populations: defining, estimating and interpreting *F*_ST_. Nat Rev Genet.

[65] Oksanen J, Blanchet FG, Kindt R, Legendre P, Minchin PR, O’hara RB (2013). Package ‘vegan’. Community Ecol Package, Vers.

