# Supplementary material for: Soil bacterial populations are shaped by recombination and gene-specific selection across a grassland meadow
Source: ISME J. 2020 Apr 23;14(7):1834–46. doi: 10.1038/s41396-020-0655-x (PMC7305173; doi:10.1038/s41396-020-0655-x)
Supplement: Supplementary file 1 — Supplementary Figure Legends [file 41396_2020_655_MOESM1_ESM.docx]

**Supplementary Information**

**Supplementary Figure S1 The impact of coverage on the number of SNPs called in each sample.** Each point is a sample-genome mapping pairing, with the number of SNPs called per sample plotted against the mean coverage of the genome in that sample, the mean nucleotide diversity of the genome in that sample, and the mean nucleotide diversity times the coverage, demonstrating that at low coverages the number of SNPs called is a function of nucleotide diversity and coverage.

**Supplementary Figure S2 Impact of subsampling genomic coverage on nucleotide diversity.** Reads mapping to each representative genome were subsampled to an exactly even coverage at every position, and mean nucleotide diversity for each gene was calculated at a range of coverages from 5x to 100x.

**Supplementary Figure S3 Linkage disequilibrium decay over genomic distance for all 19 species in this study.** Each block point represents a mean of linkage for SNPs at that genomic distance, divided into non synonymous-nonsynonymous linkages, nonsynonymous-synonymous linkages, and synonymous-synonymous linkages. The size of each block point represents the number of SNPs that went into calculating the mean.

**Supplementary Figure S4 Biallelic haplotype counts for each species.** The percentage of haplotype counts for each pair of biallelic sites within ~1 Kb of each other is shown for both nonsynonymous-nonsynonymous and synonymous-synoymous pairs of segregating sites.

**Supplementary Figure S5 Relationships between nucleotide diversity and relative abundance.** On the left is the relationship between the mean nucleotide diversity and relative abundances across species for the entire meadow. On the right are the relationships for each species across all of the individual samples from the meadow; linear regressions are drawn for all significant correlations (p<0.05, bonferroni corrected).

**Supplementary Figure S6 Relationship between nucleotide diversity and the N:S SNP ratio across species.** Each point represents the nucleotide diversity observed for each genome within each sample. A linear regression is shown (R^2^=0.21; p<2.2*10^-16^).

**Supplementary Figure S7 Relationship between D’ and D’_N_/D’_S._** The ratio of nonsynonymous polymorphism linkage to synonymous polymorphism linkage (D’_N_/D’_S_) versus mean D' for all bacterial populations studied. A linear regression model is shown (F-statistic: 15.6; Adjusted R-squared: 0.45, p=0.001).

**Supplementary Figure S8 Cross-population correlations between means of all population genetics summary statistics in this study.** In each box, the pearson correlation coefficient between two metrics is shown. Only correlation coefficients with a p-value <0.01 are shown.

**Supplementary Figure S9 Mean FST values for every species across the meadow.** The mean per-gene FST values for each species between each of the three block comparisons is shown.

**Supplementary Figure S10 Gene FST along the genome for every species across the meadow.** Per-gene FST values are plotted in ORF order on contigs. The title of each plot shows the species name and the block vs block comparison values being plotted. Each point is a gene, and the size of the point increases with the number of segregating sites in that each. Genes are colored in red if they are part of a locus with mean FST greater than 2.5 times the standard deviation of the genomic average.

**Supplementary Table S1 Completeness and contamination statistics for all population replicate genomes included in this study.**

**Supplementary Table S2 Key population genetics summary statistics in this study.**

**Supplementary Table S3 ComEC annotations for each species.**

**Supplementary Table S4 FST 95% Confidence intervals for each species.**

**Supplementary Table S5 Coverage values of highly differentiated loci.**

**Supplementary Table S6 Annotations of genes within highly differentiated loci.**
