# Supplementary figures and images for: Soil bacterial populations are shaped by recombination and gene-specific selection across a grassland meadow
Source: ISME J. 2020 Apr 23;14(7):1834–46. doi: 10.1038/s41396-020-0655-x (PMC7305173; doi:10.1038/s41396-020-0655-x)

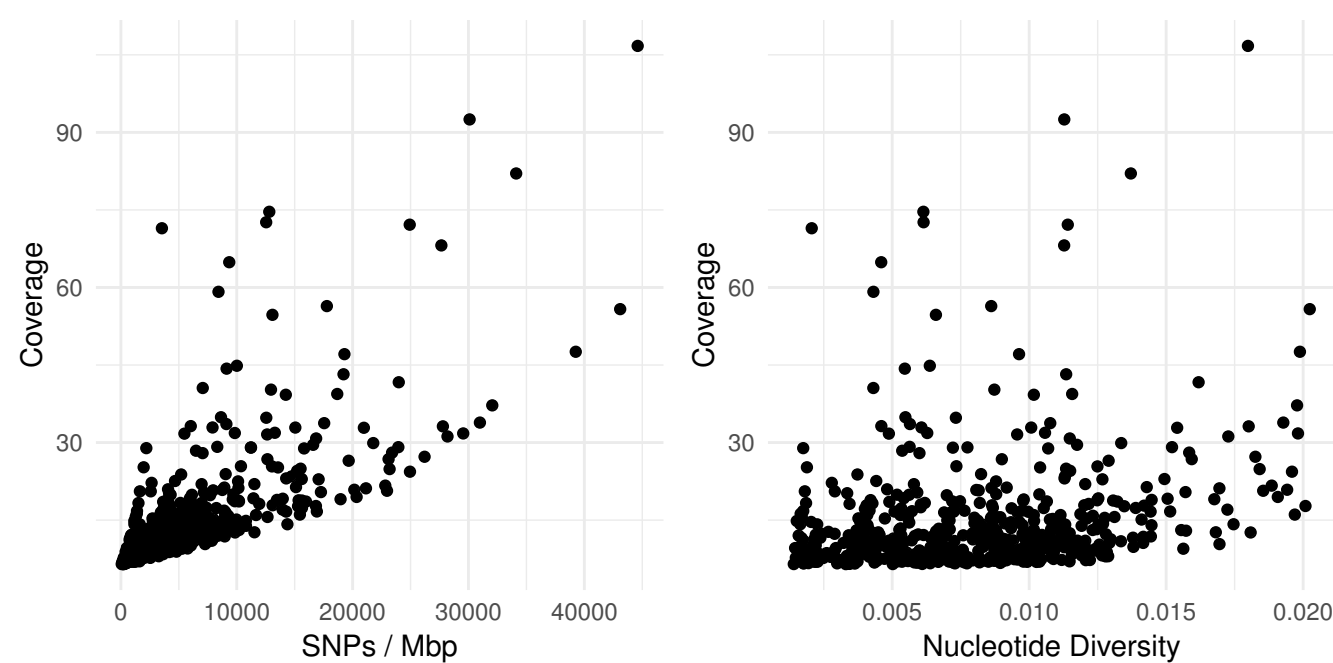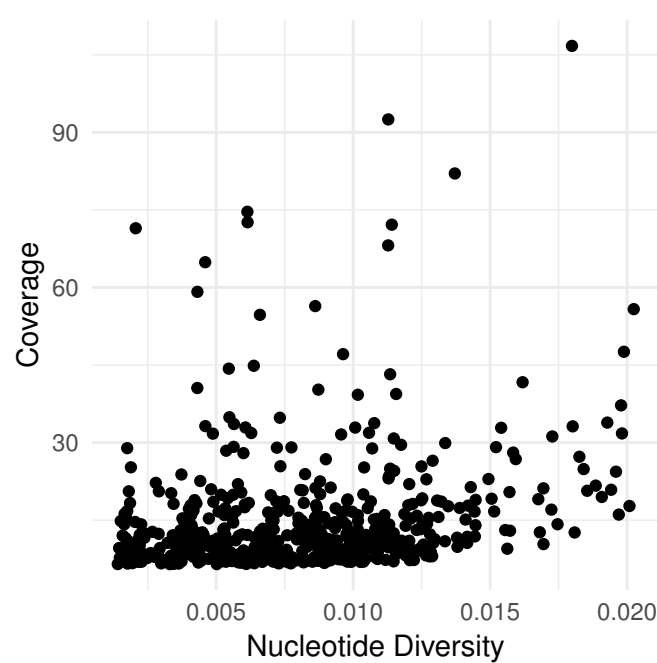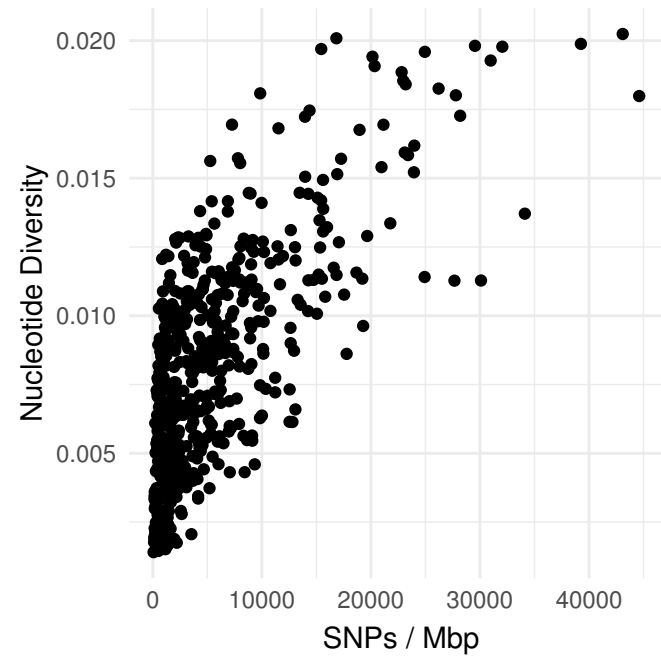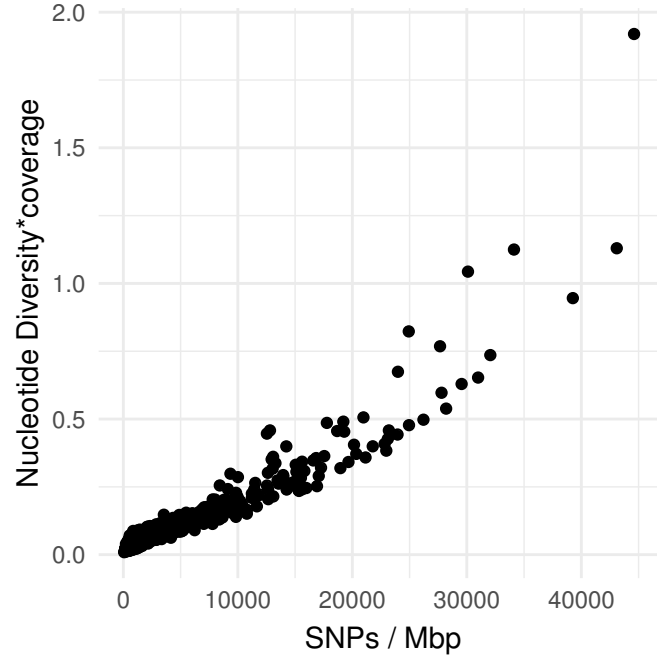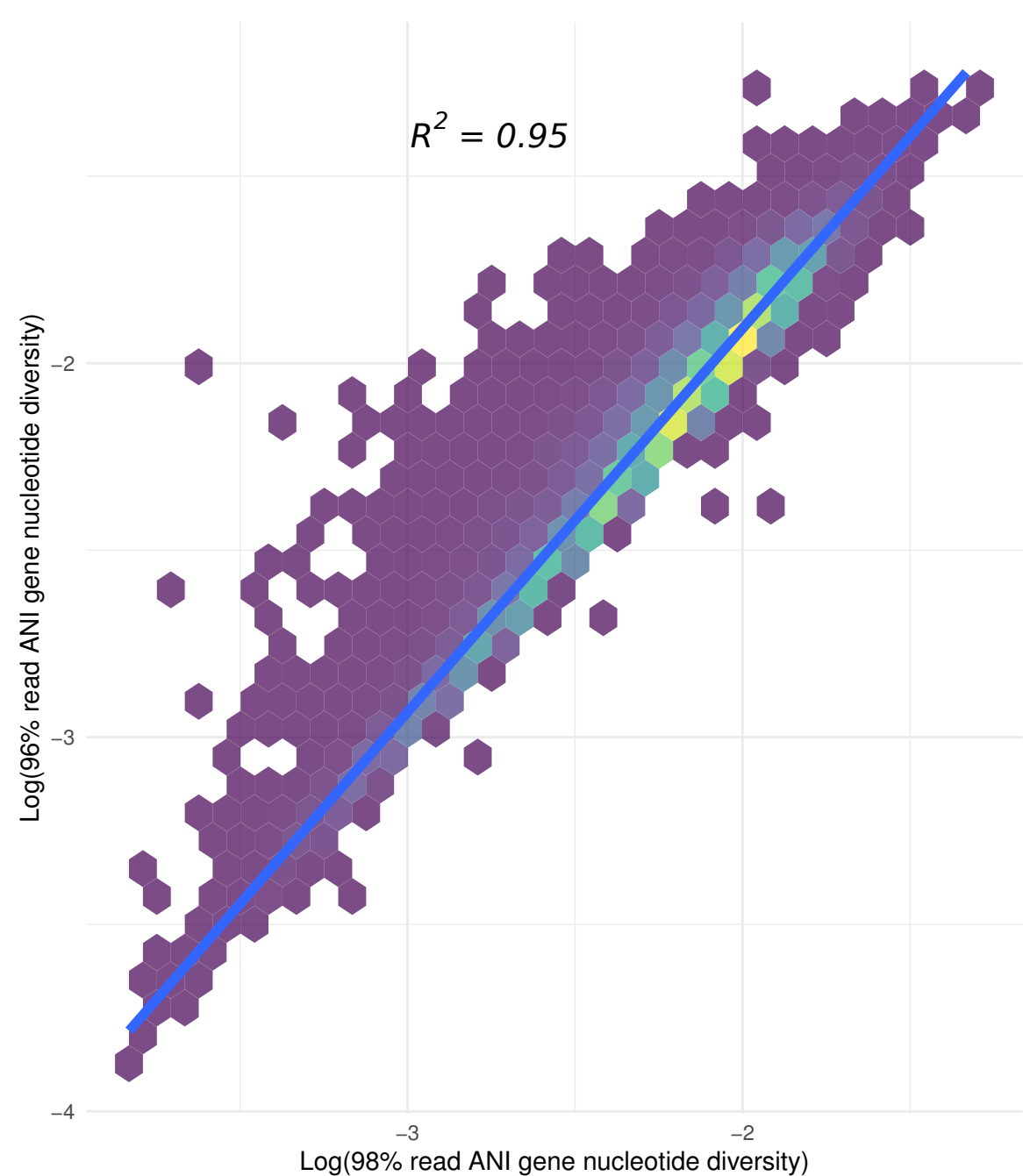

Supplement: Supplementary file 2 — Supplementary Figure S1 [file 41396_2020_655_MOESM2_ESM.pdf]

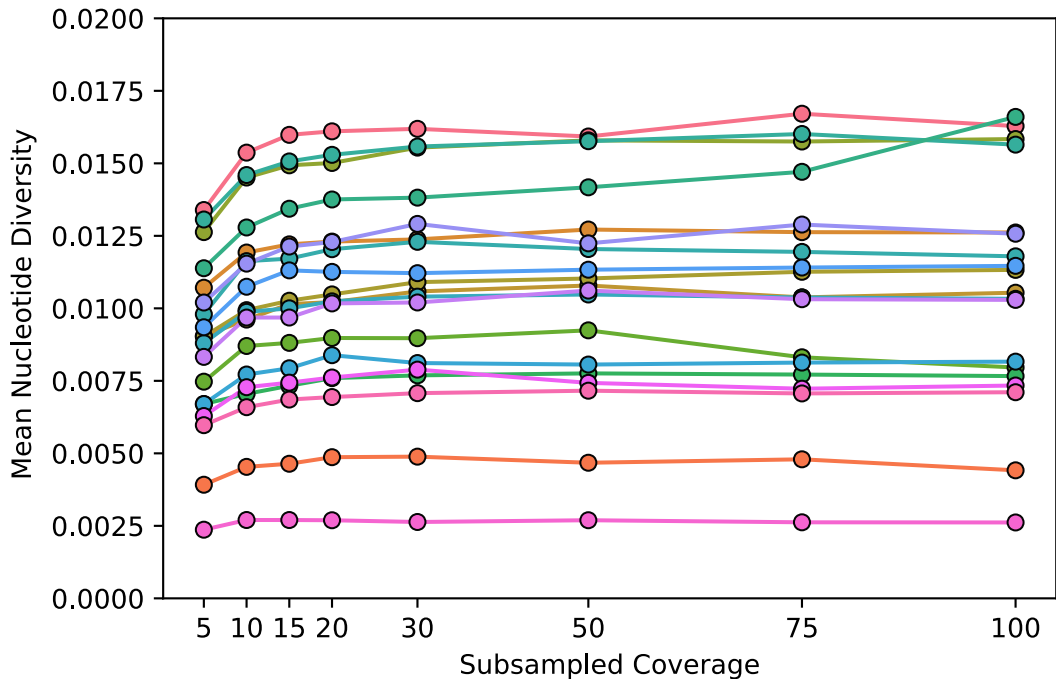

Supplement: Supplementary file 3 — Supplementary Figure S2 [file 41396_2020_655_MOESM3_ESM.pdf]

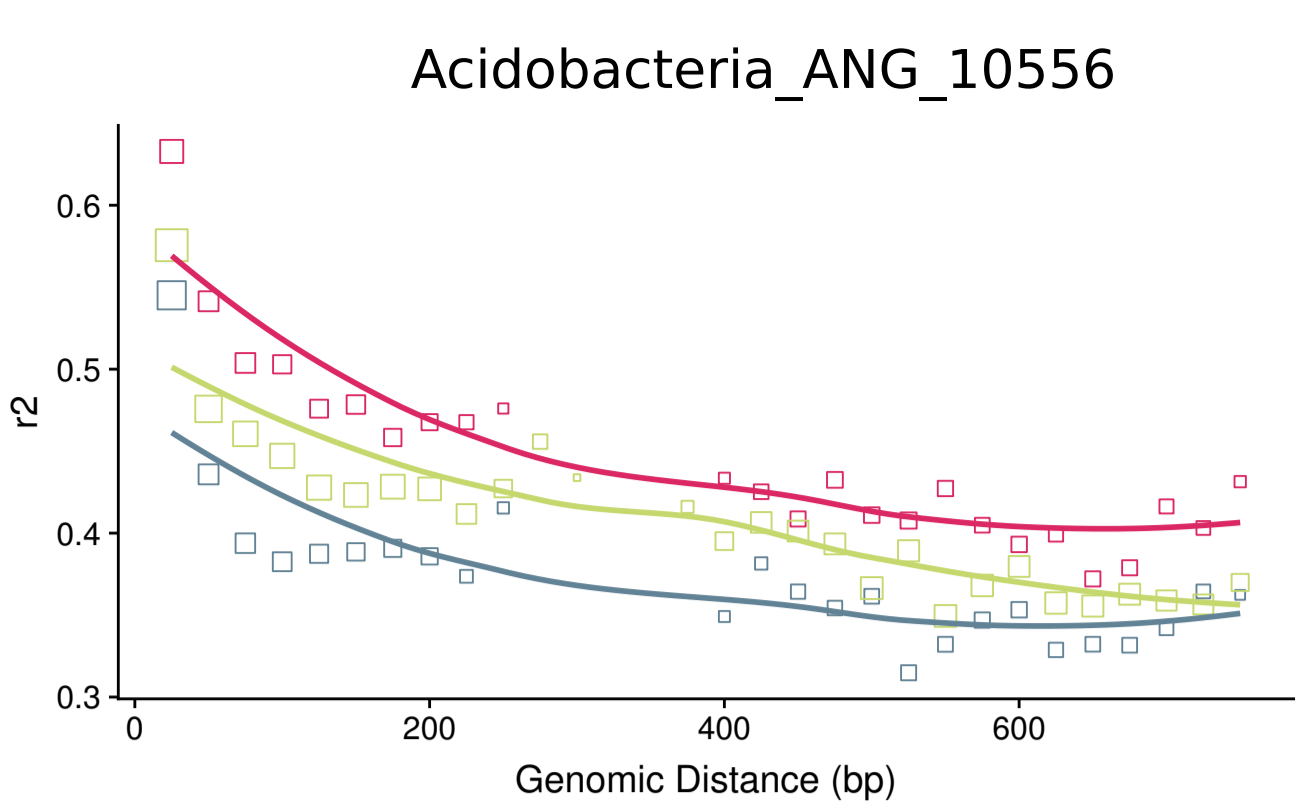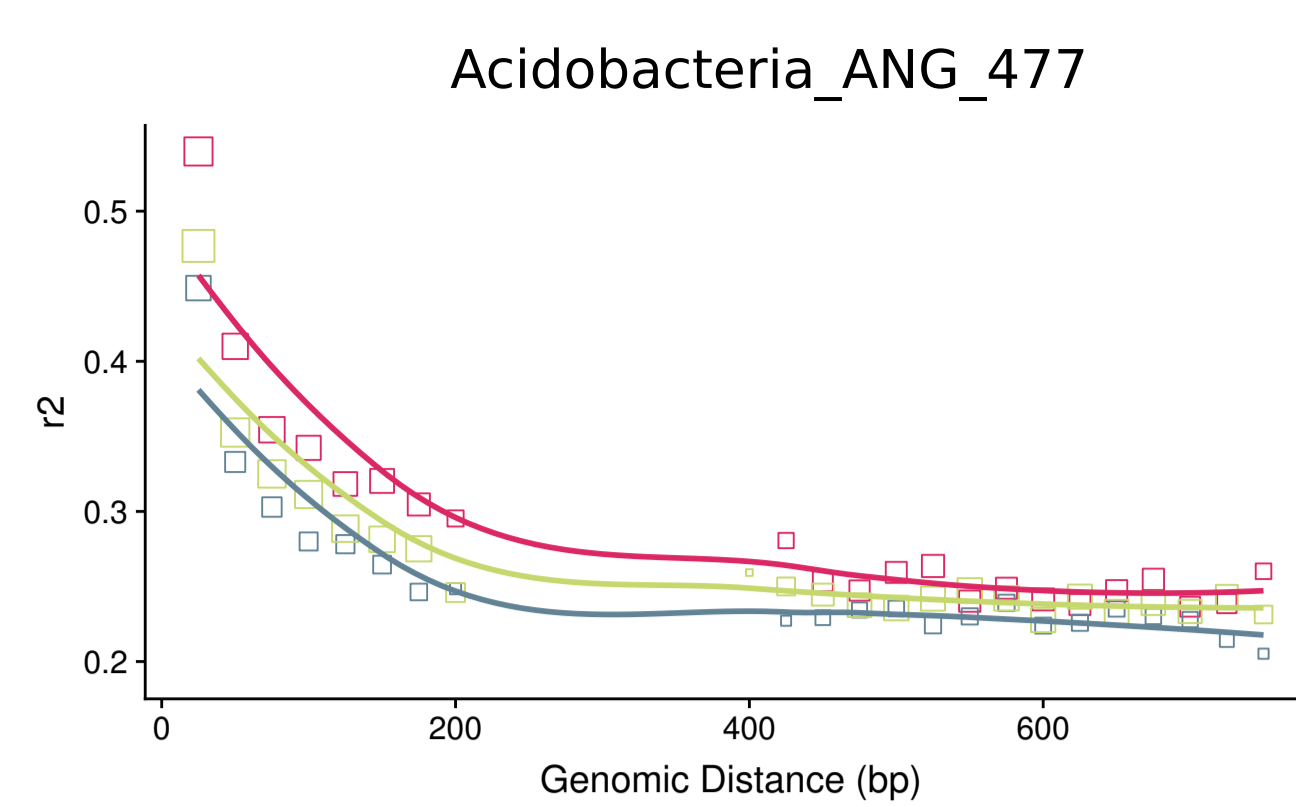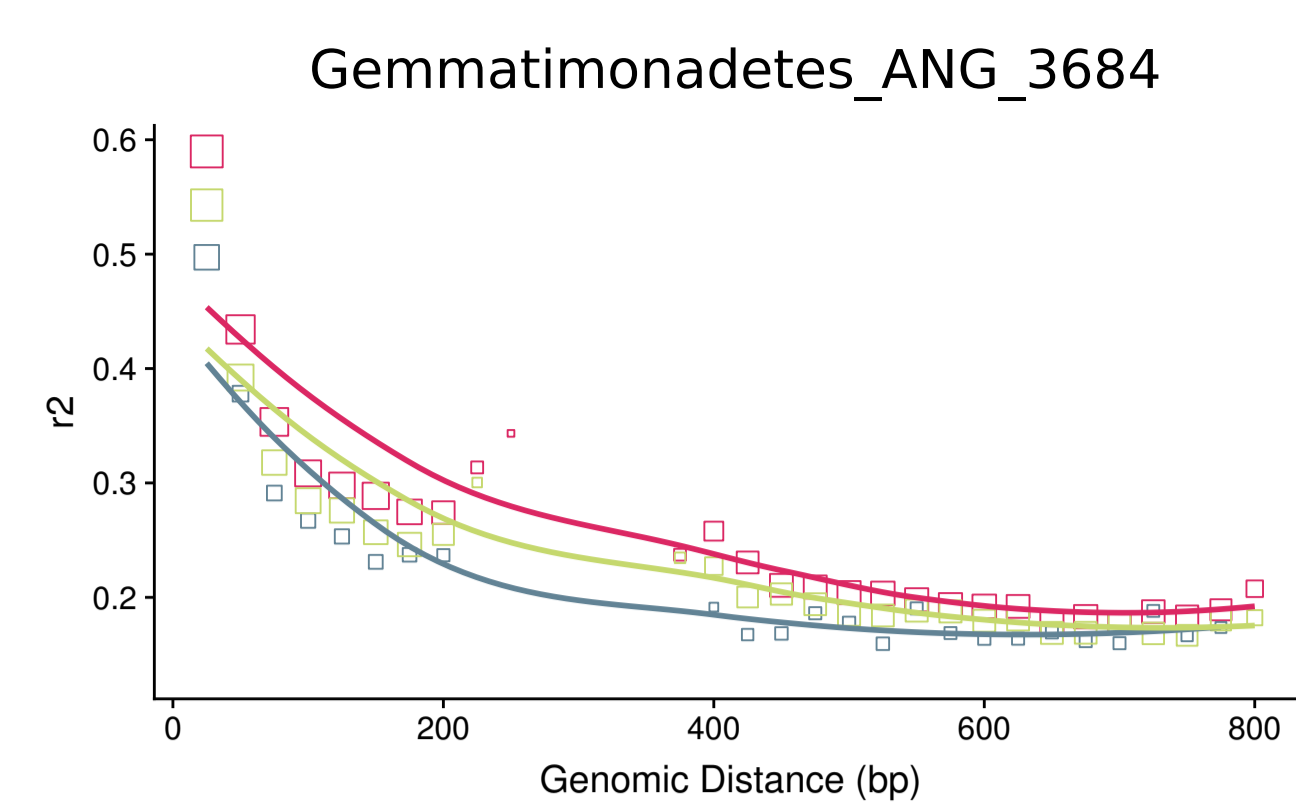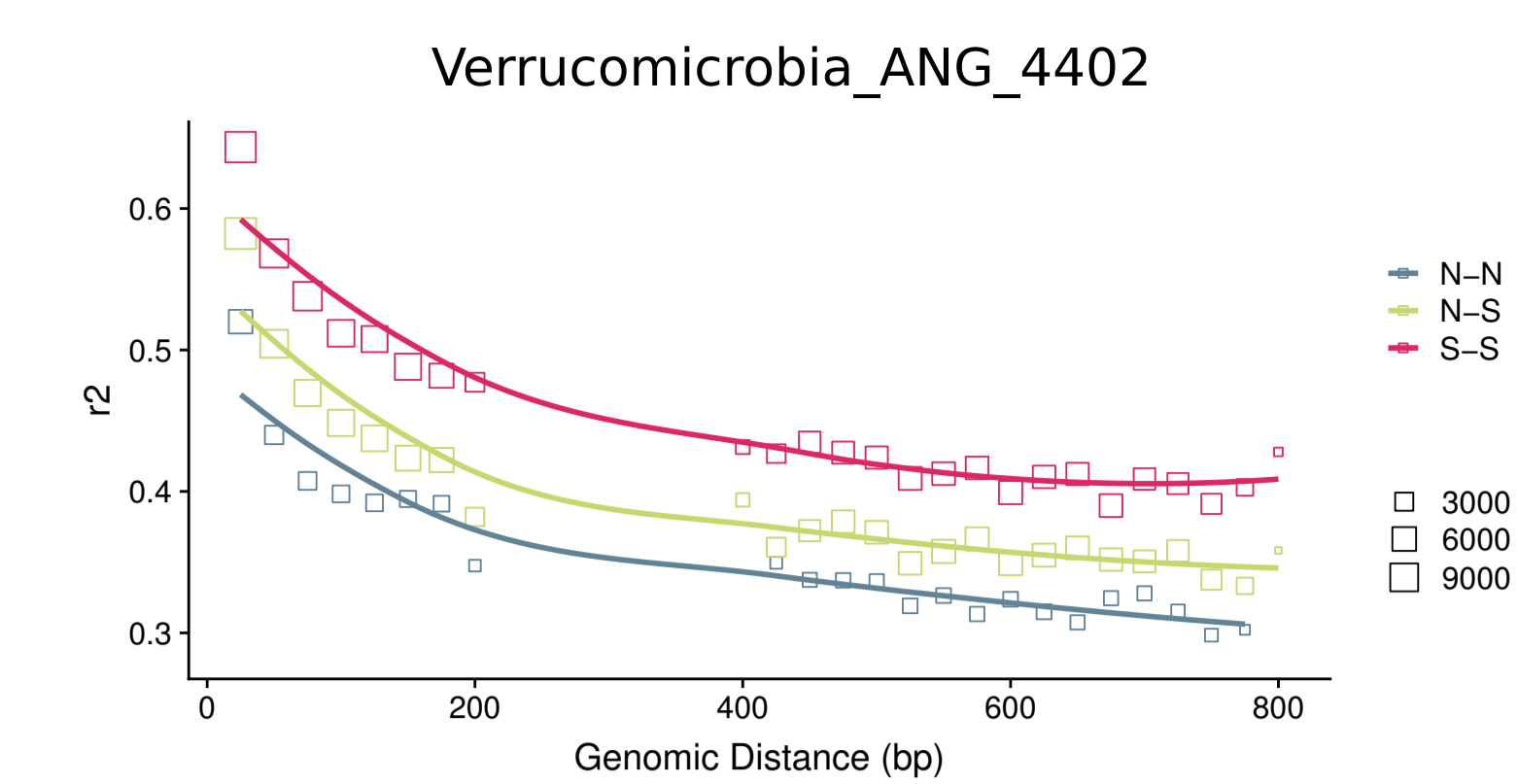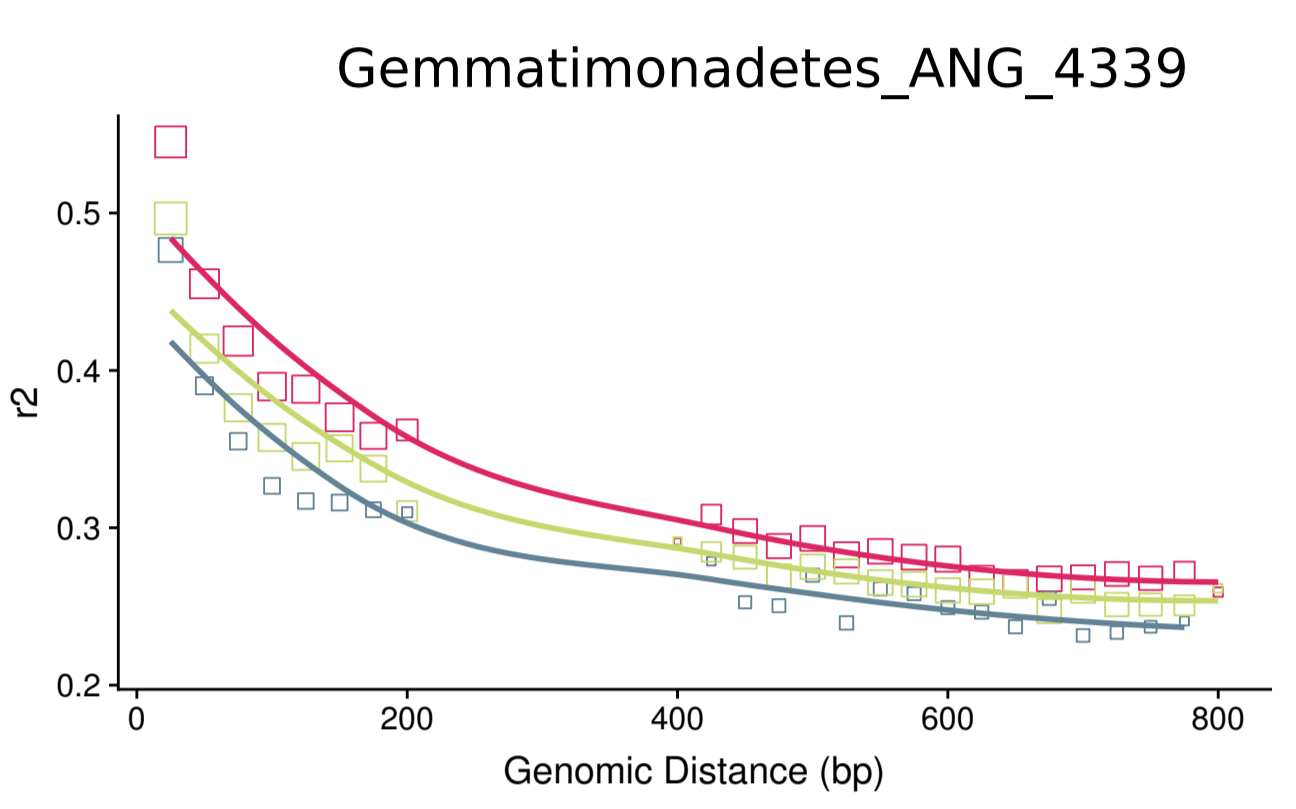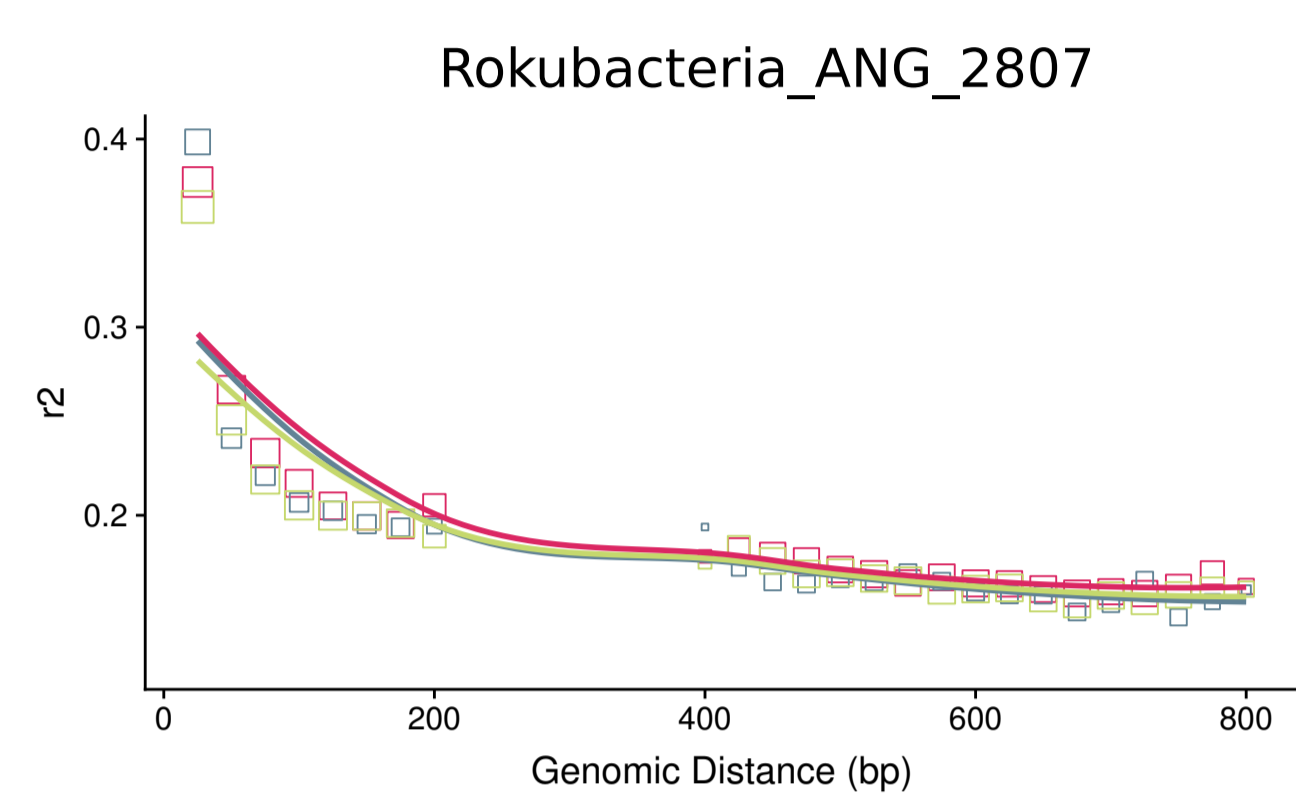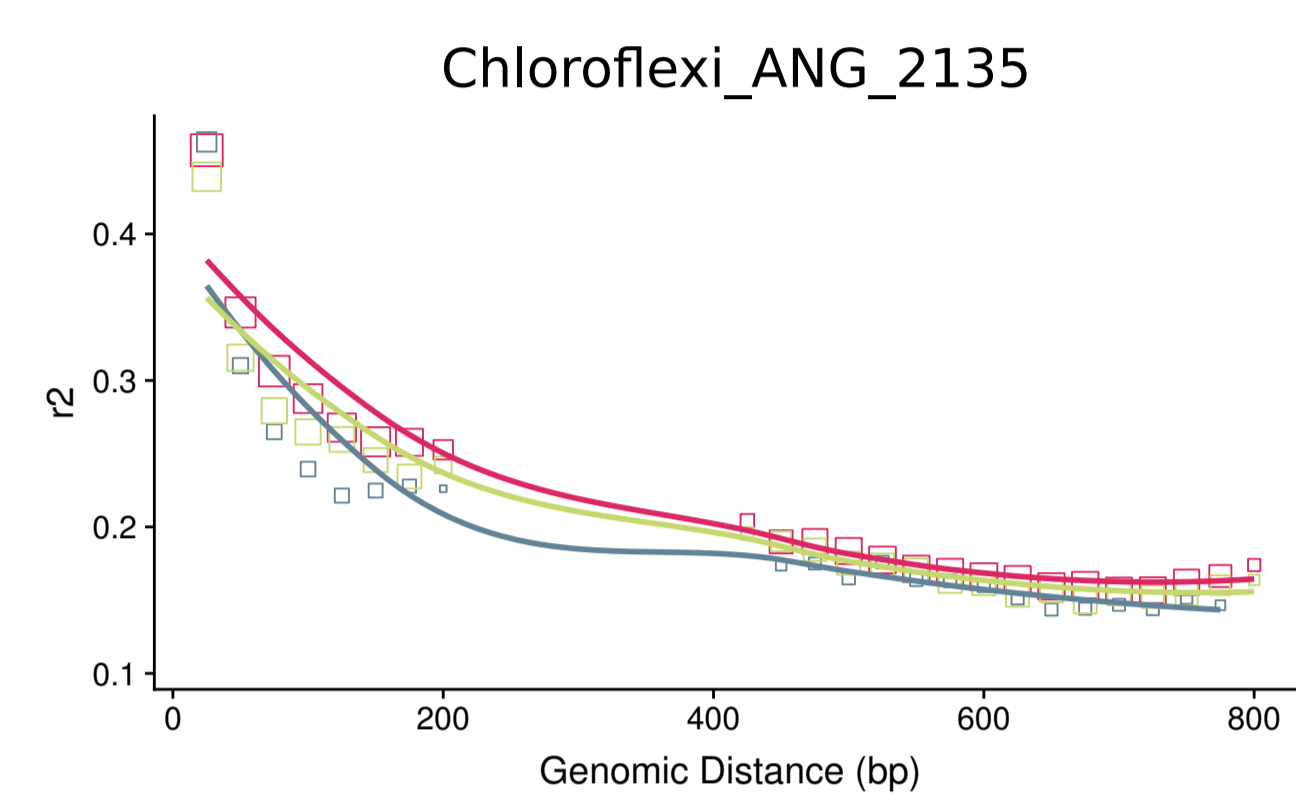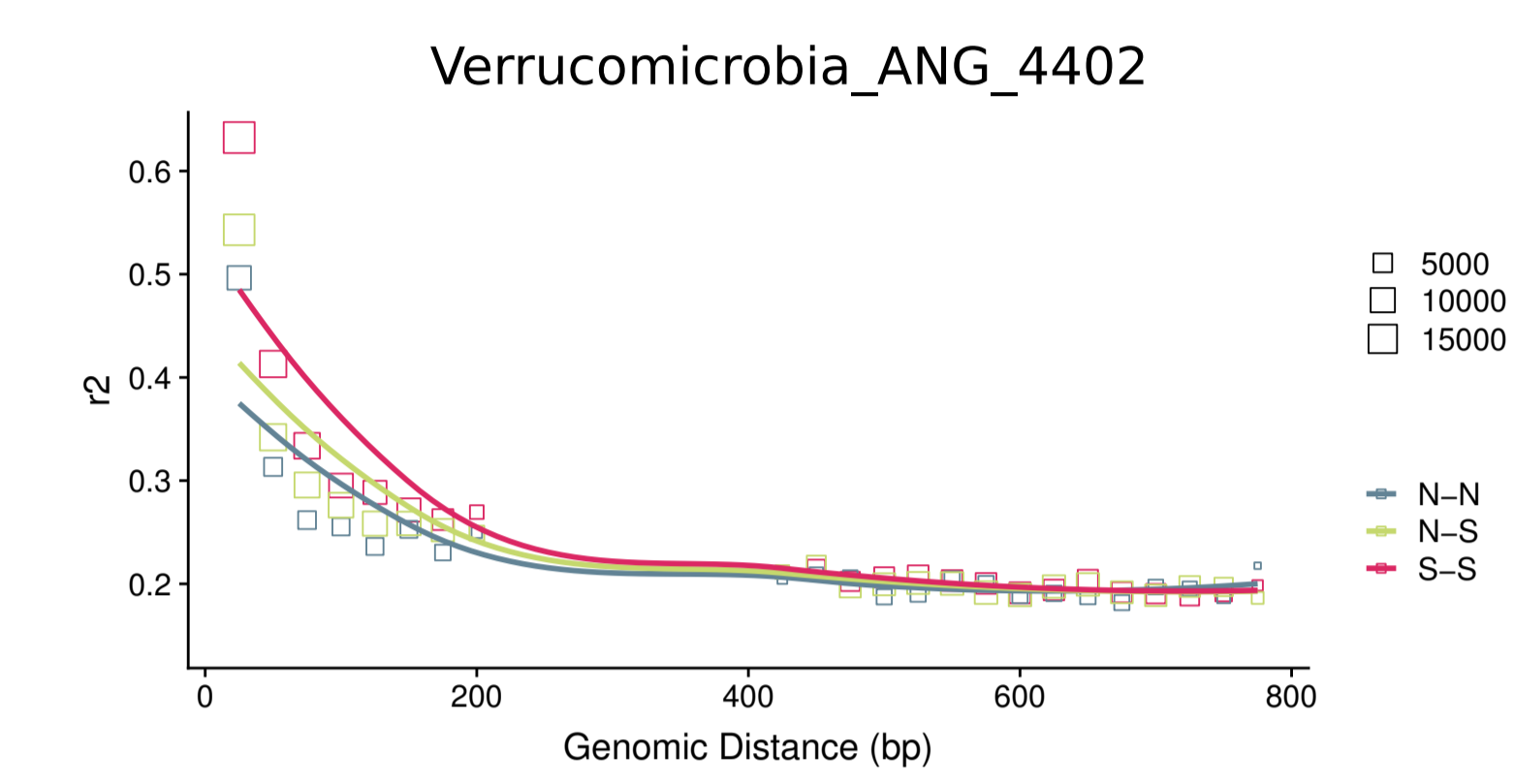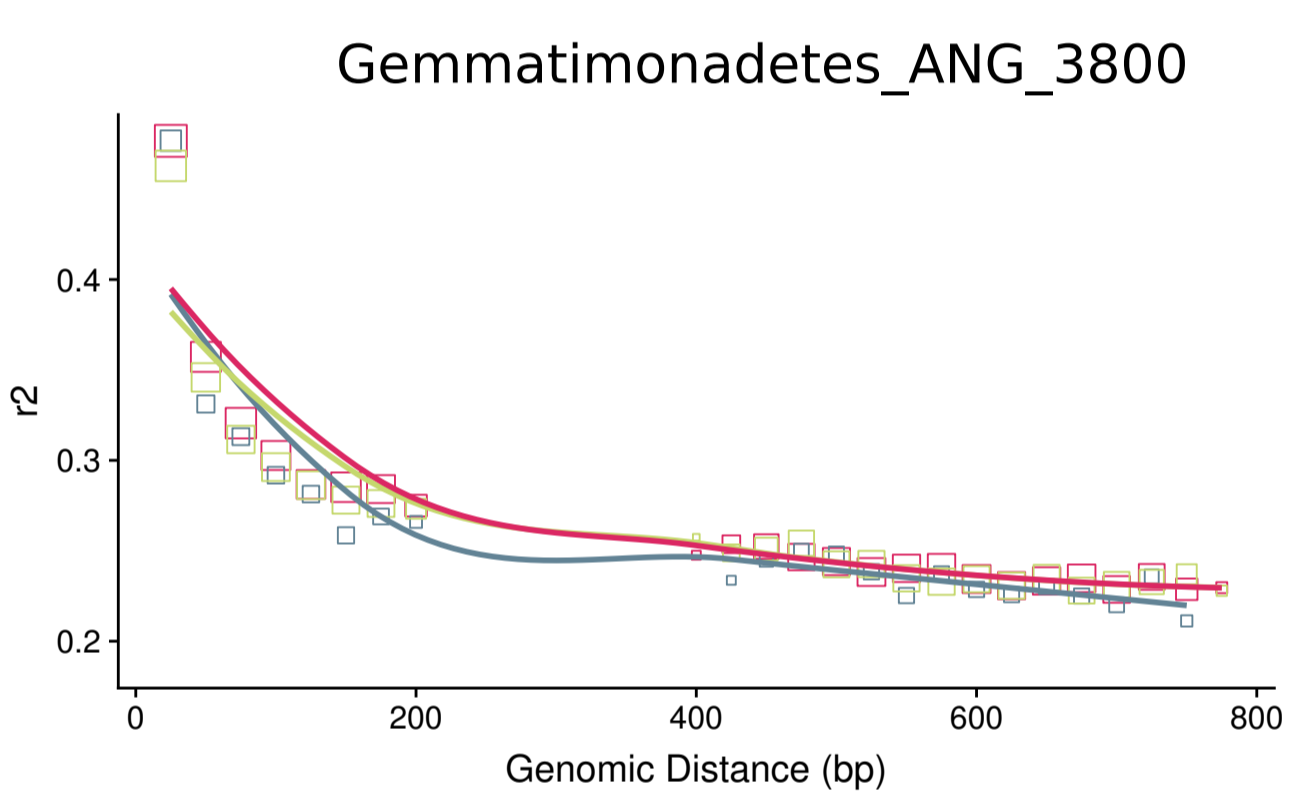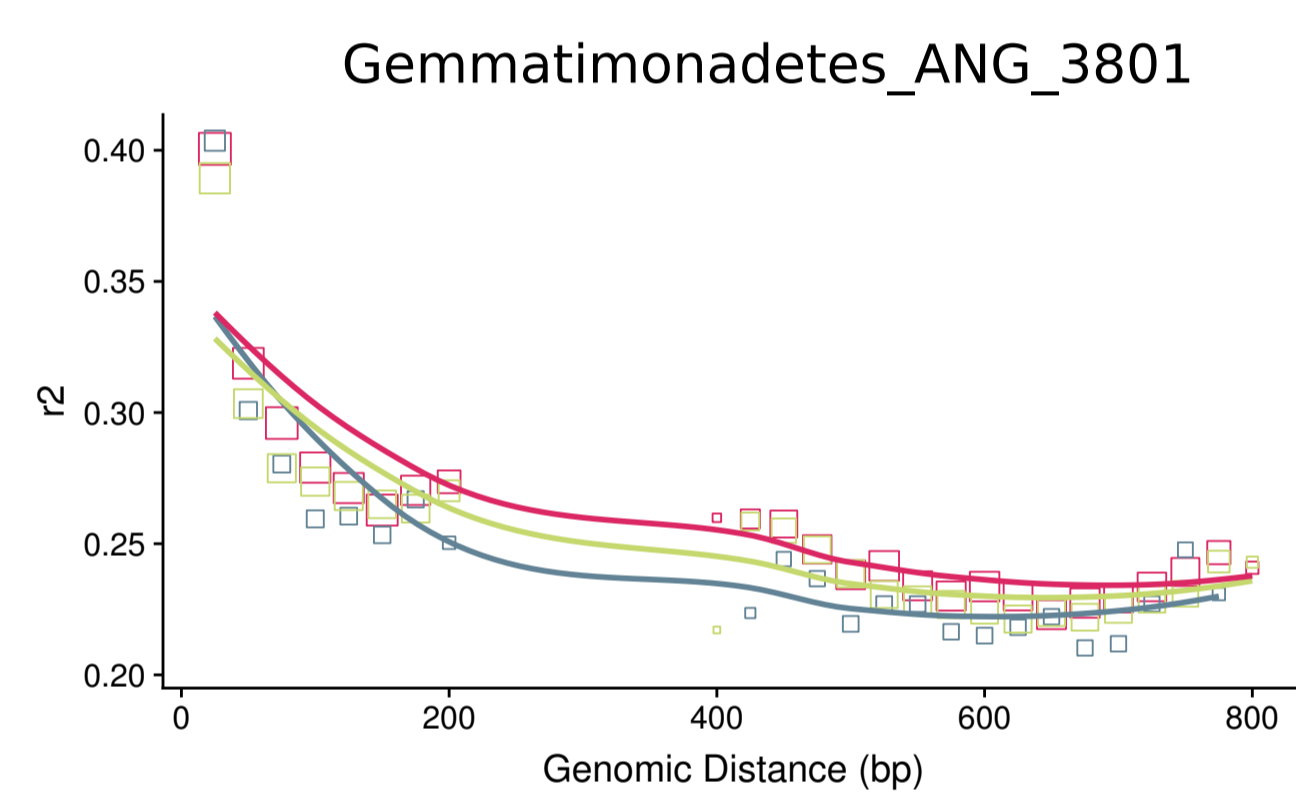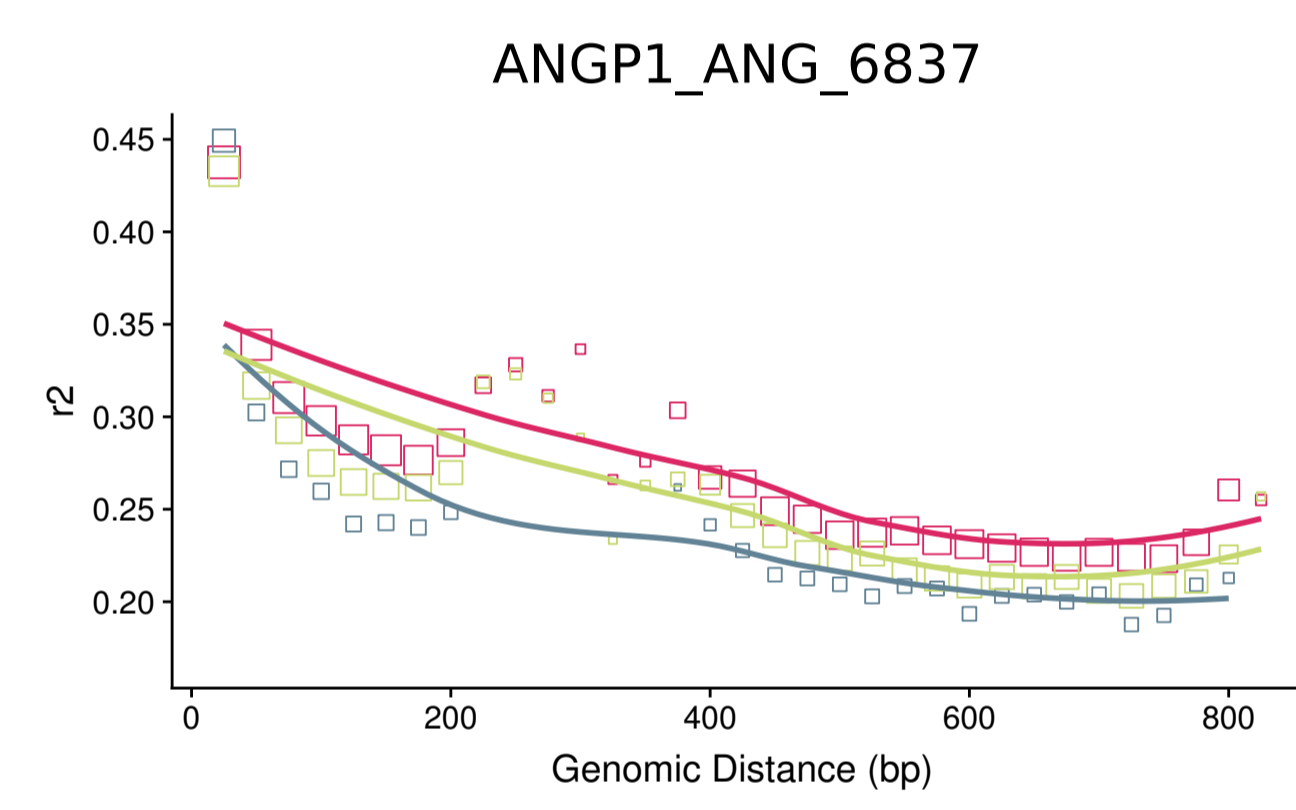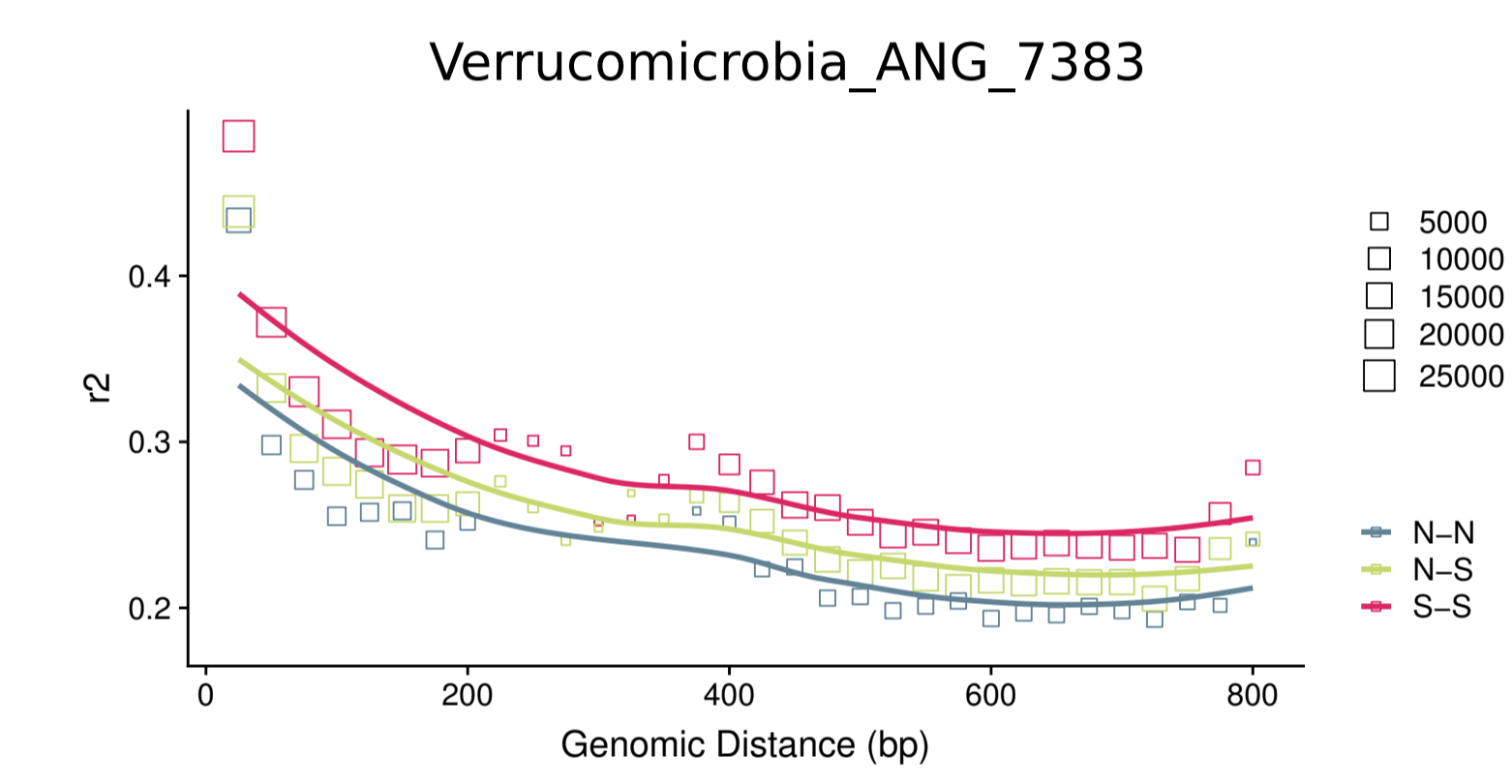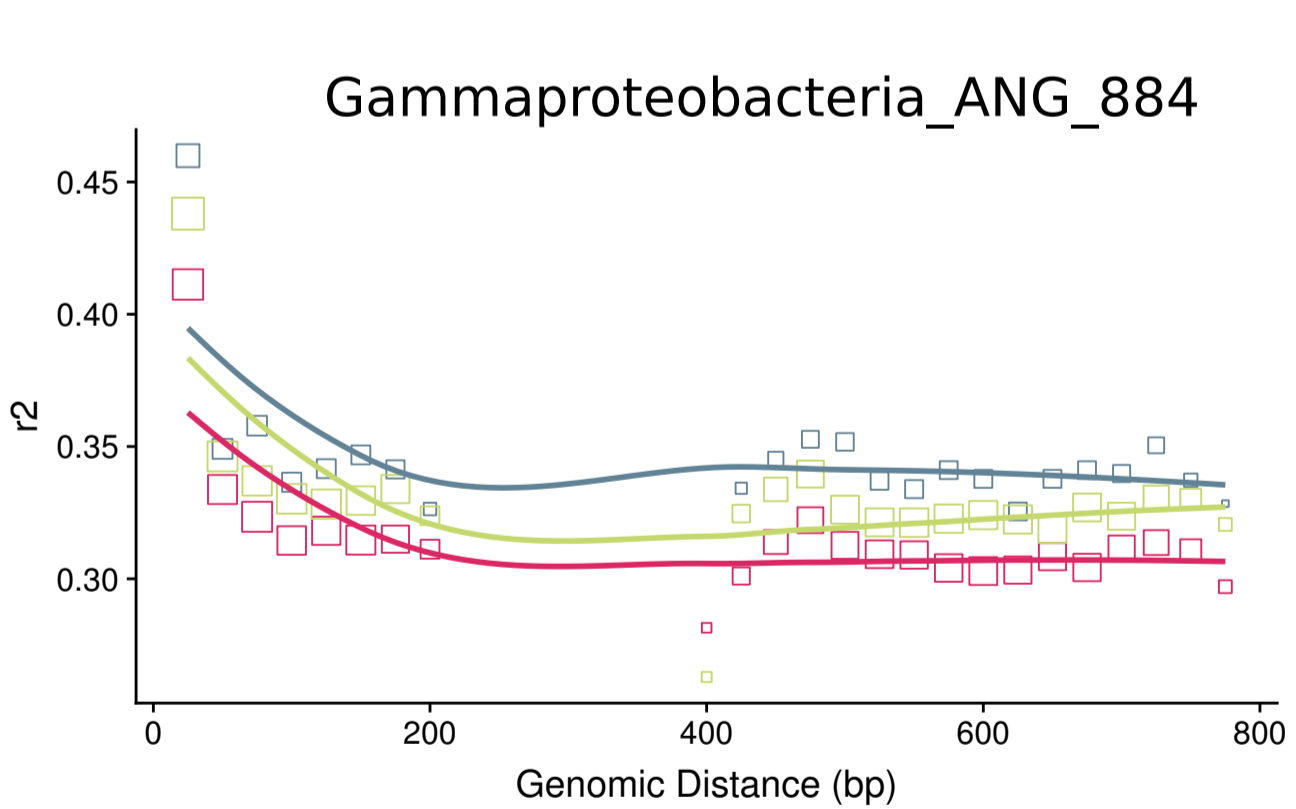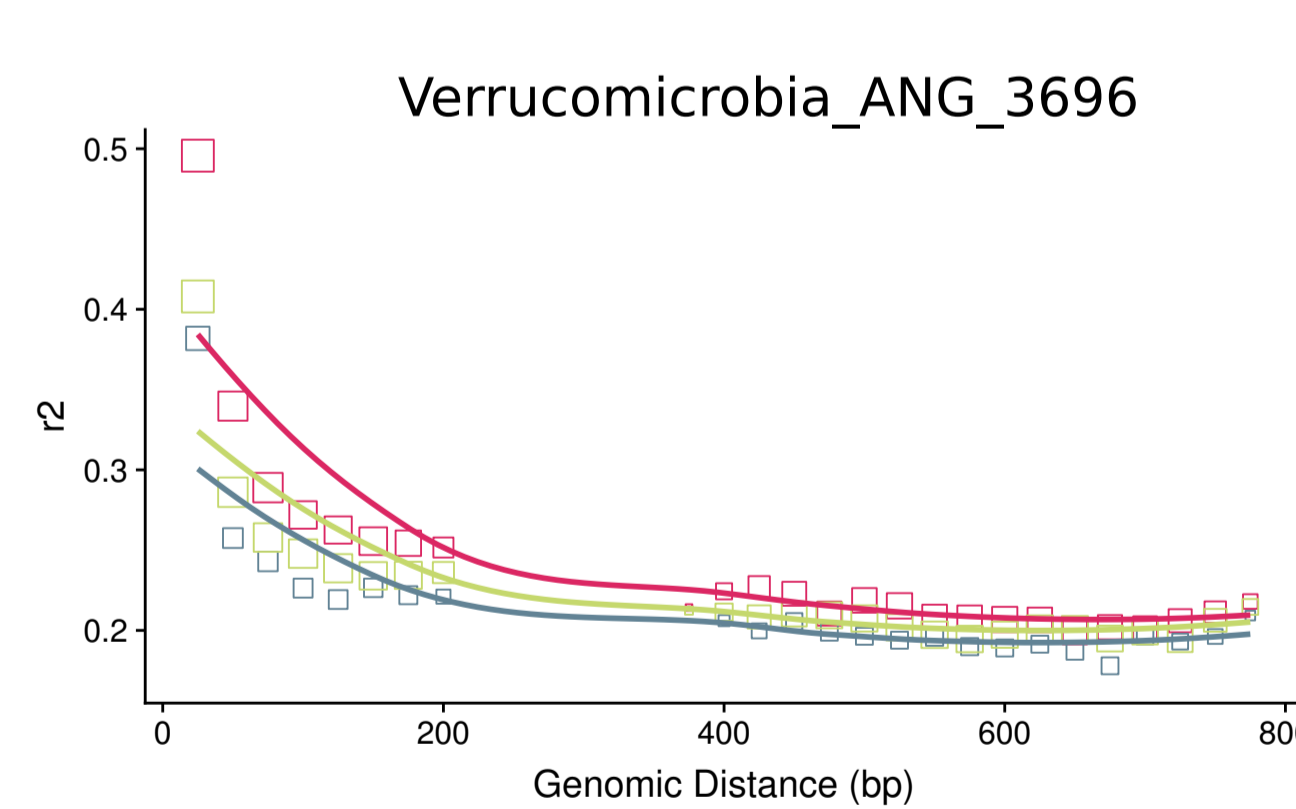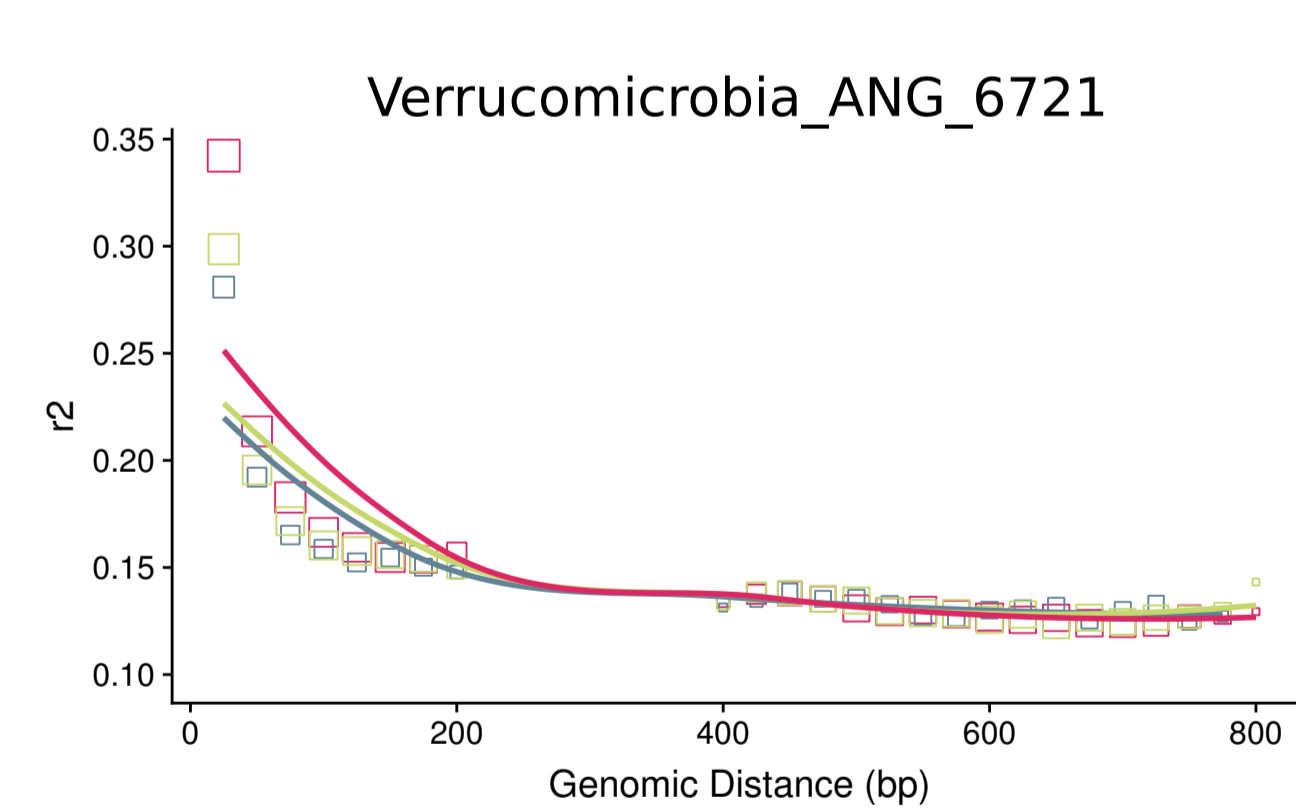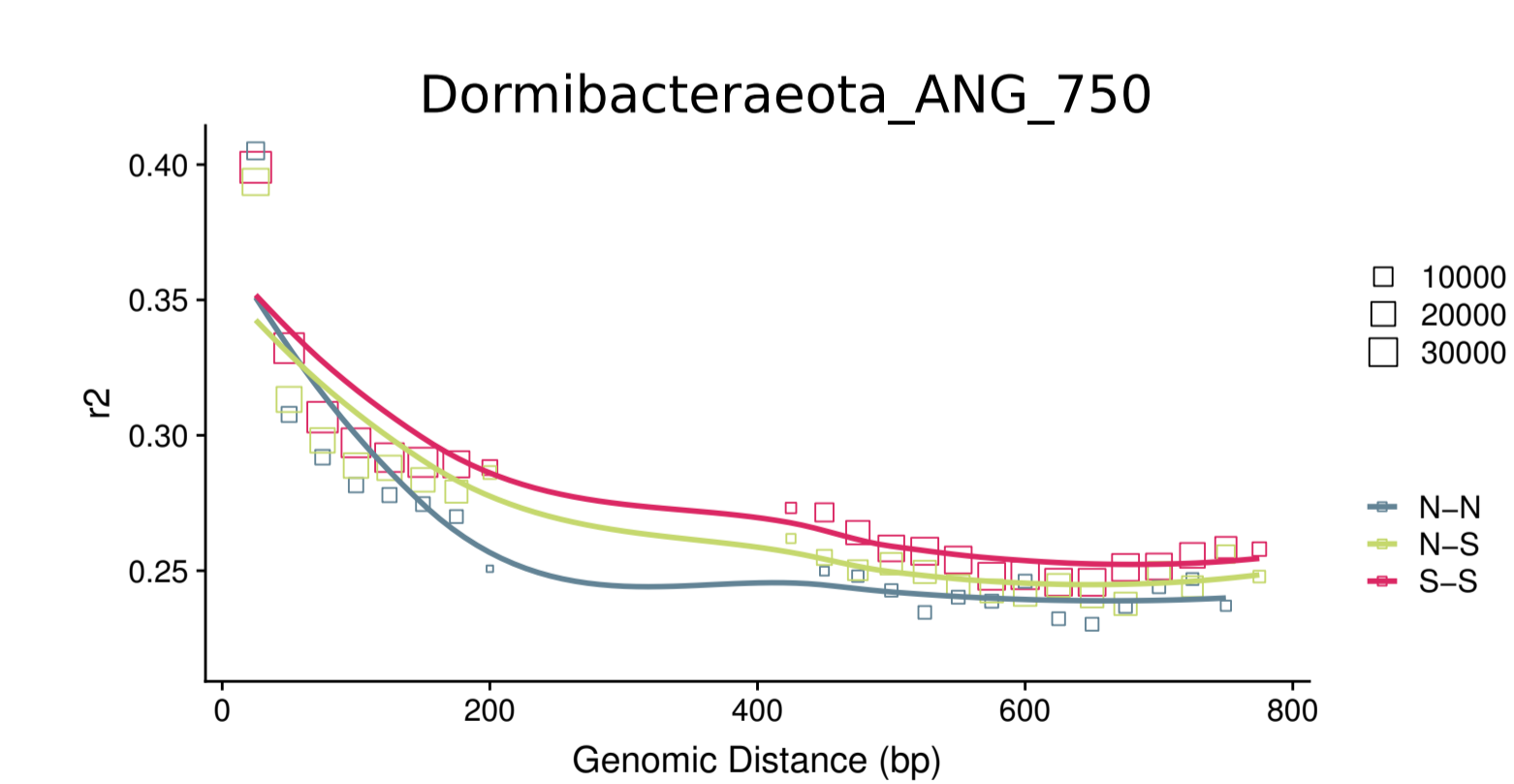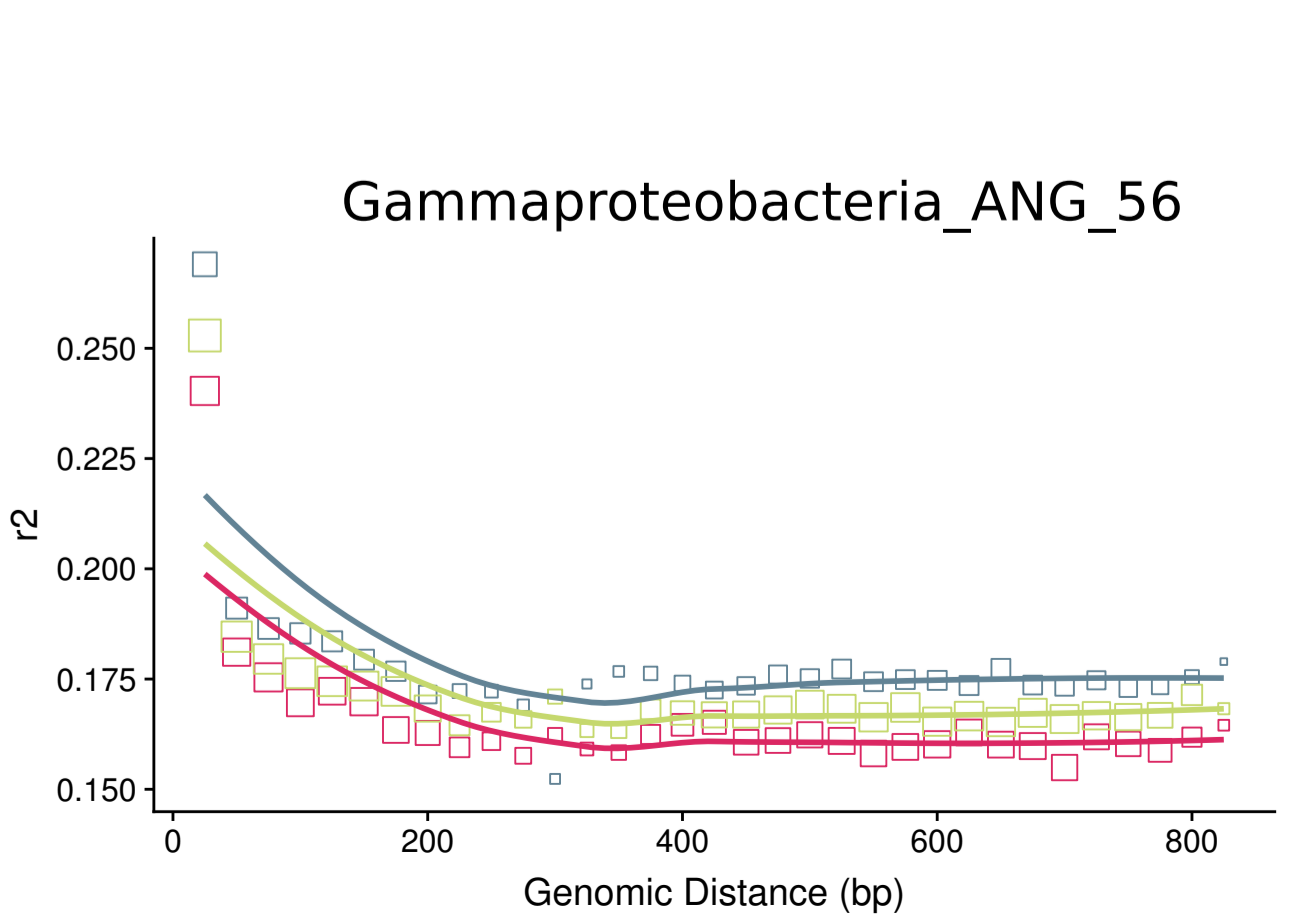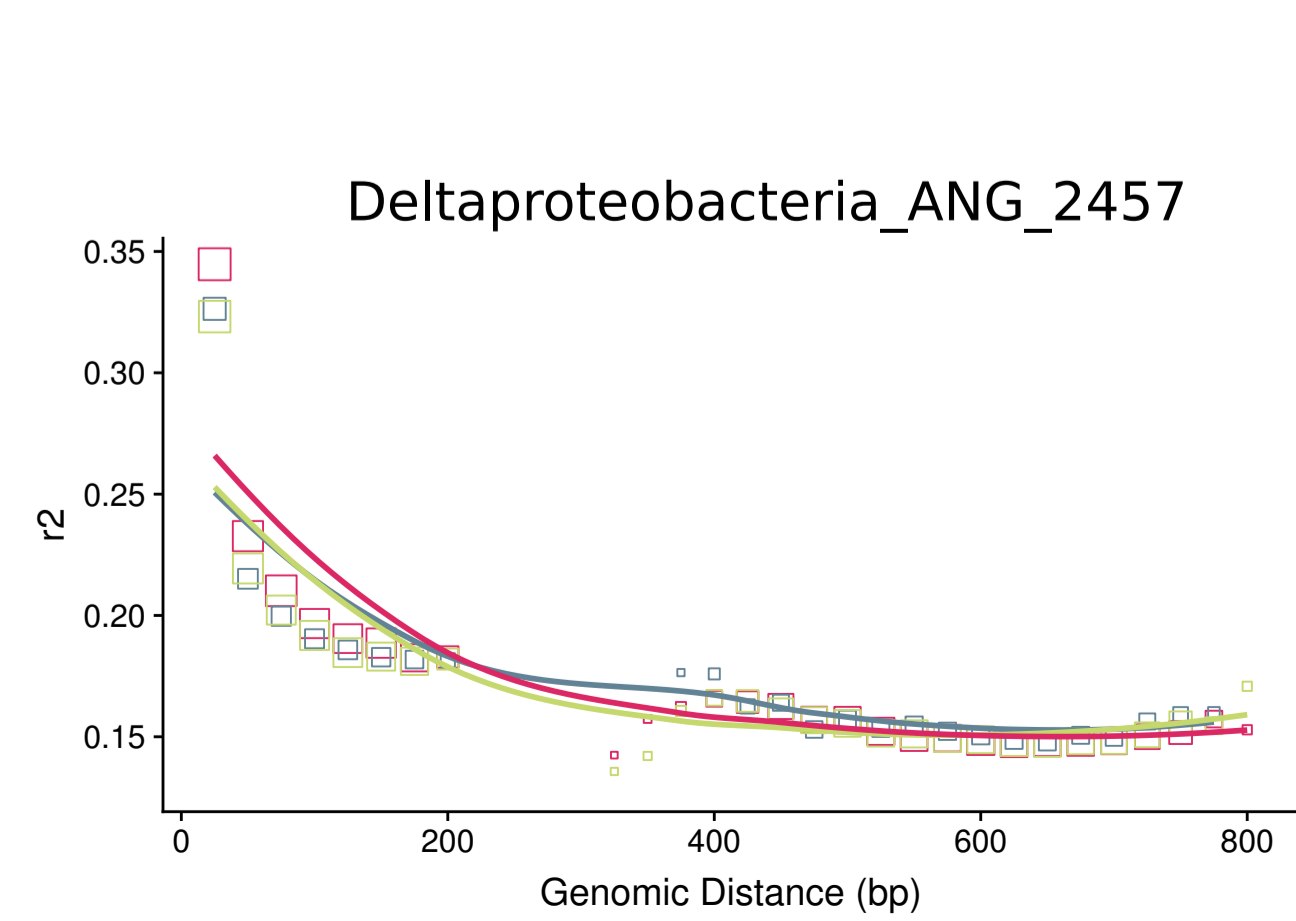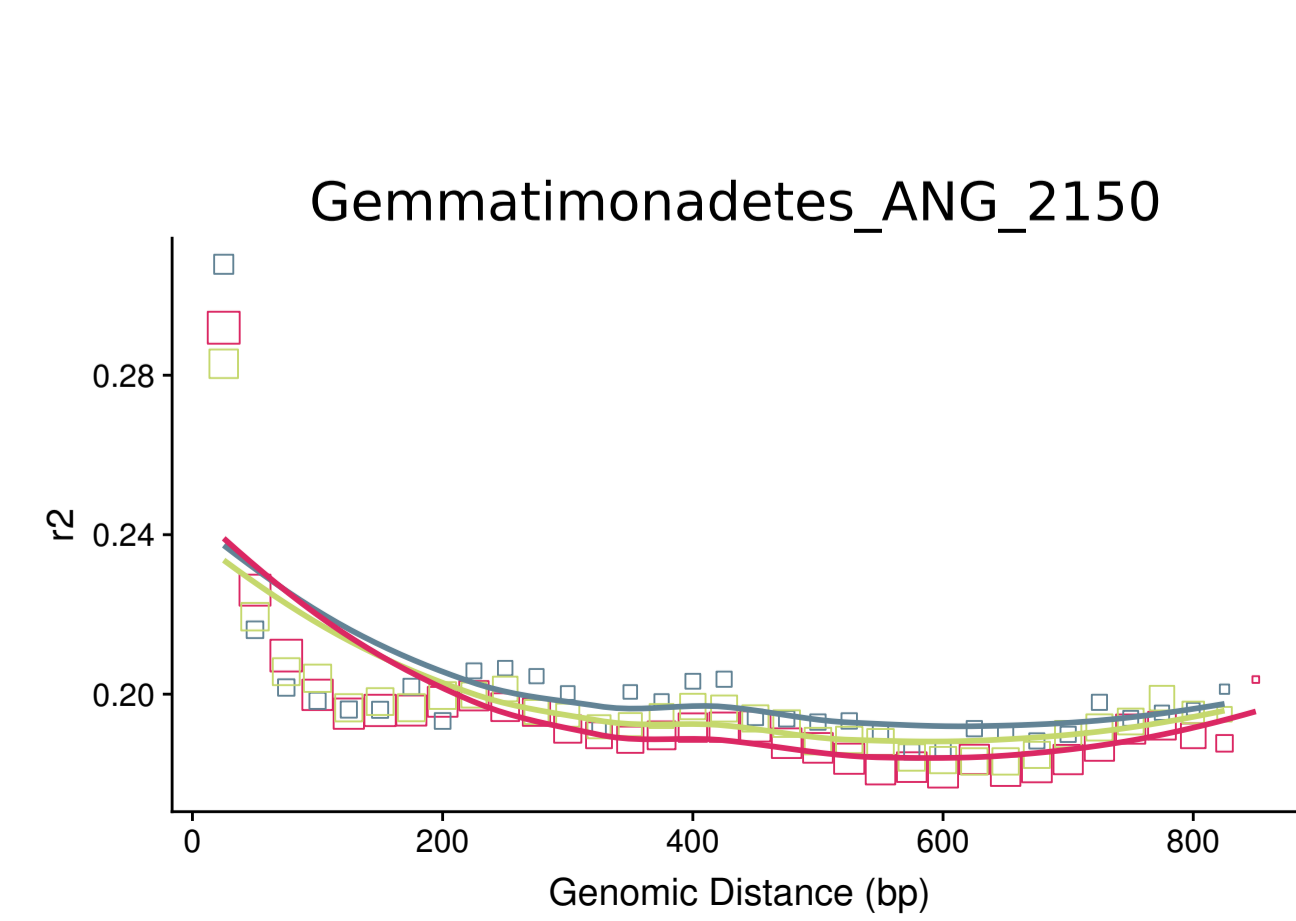

Supplement: Supplementary file 4 — Supplementary Figure S3 [file 41396_2020_655_MOESM4_ESM.pdf]

N-N mutations

S-S mutations

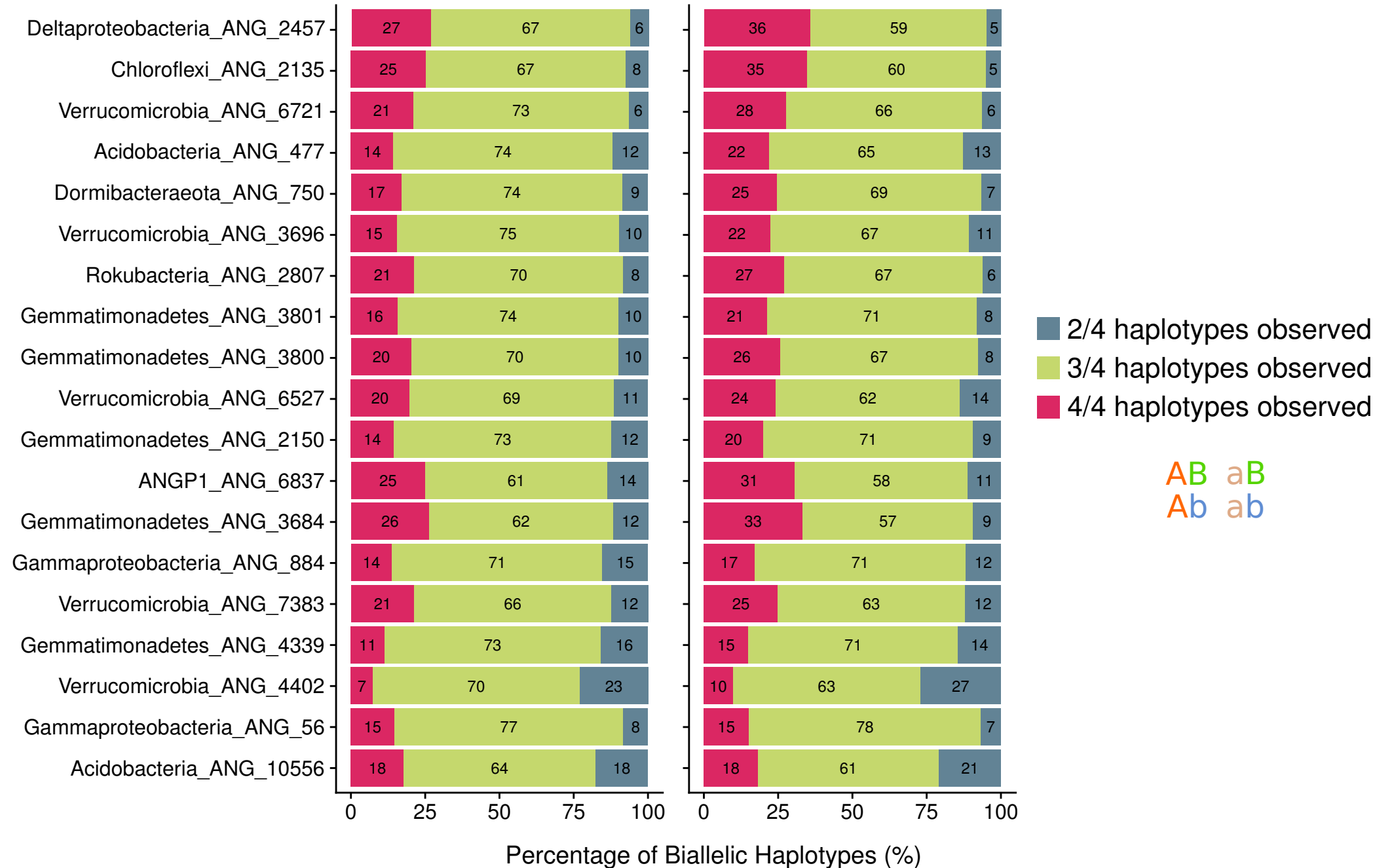

Supplement: Supplementary file 5 — Supplementary Figure S4 [file 41396_2020_655_MOESM5_ESM.pdf]

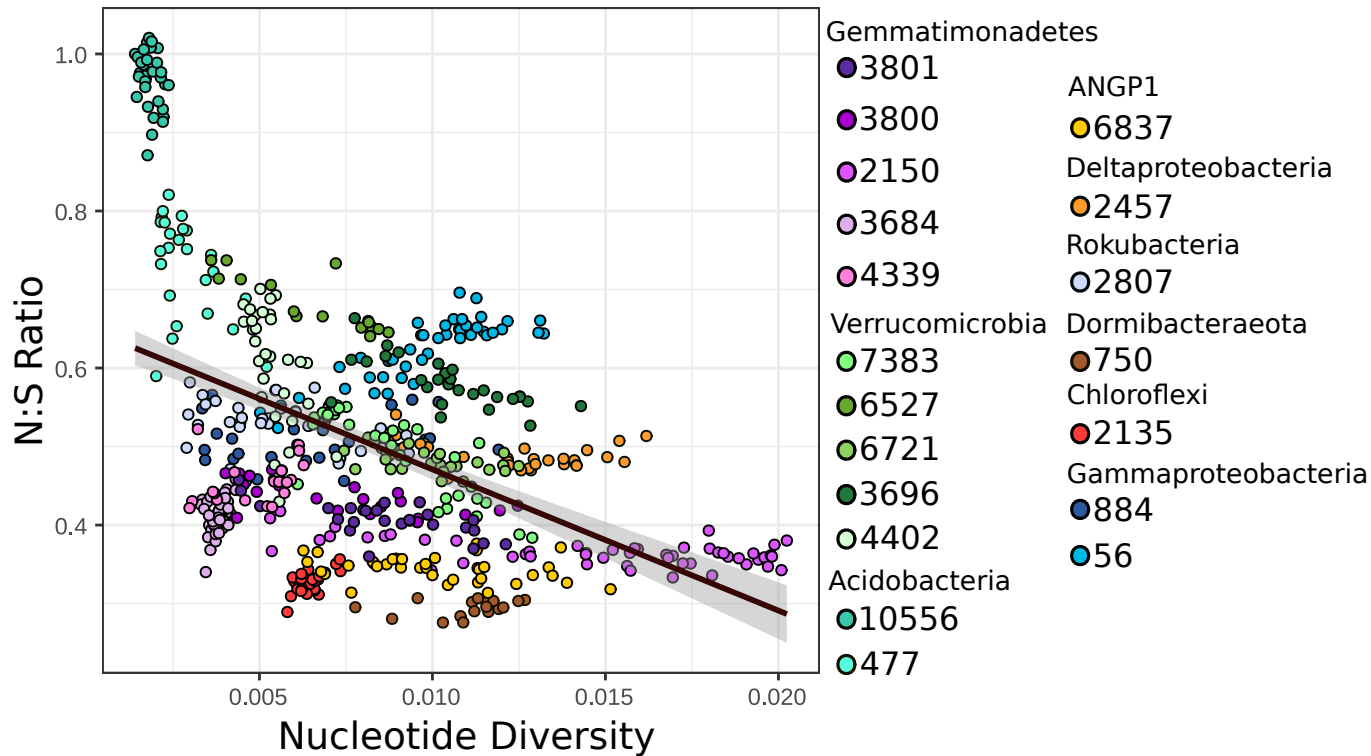

Supplement: Supplementary file 7 — Supplementary Figure S6 [file 41396_2020_655_MOESM7_ESM.pdf]

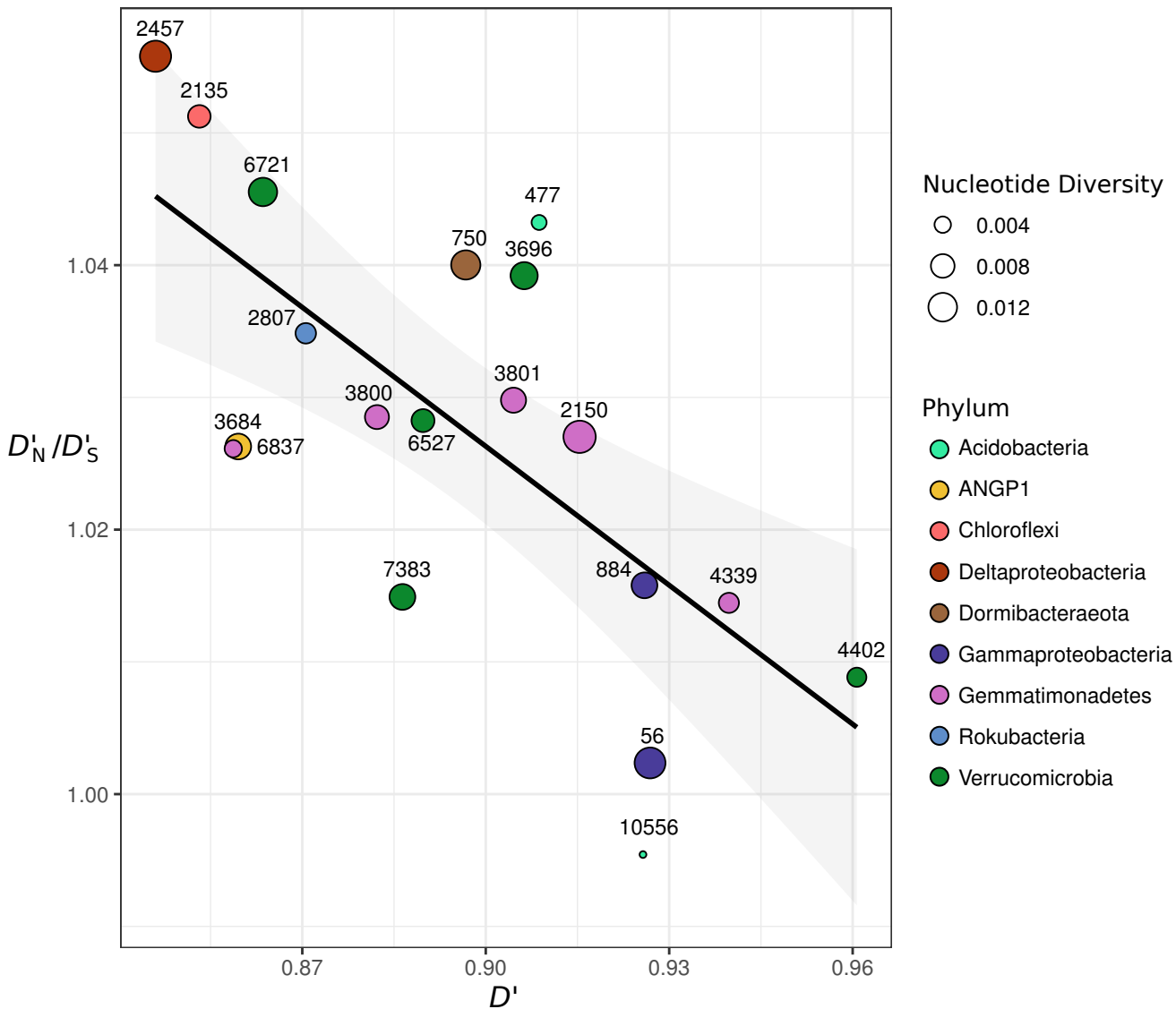

Supplement: Supplementary file 8 — Supplementary Figure S7 [file 41396_2020_655_MOESM8_ESM.pdf]

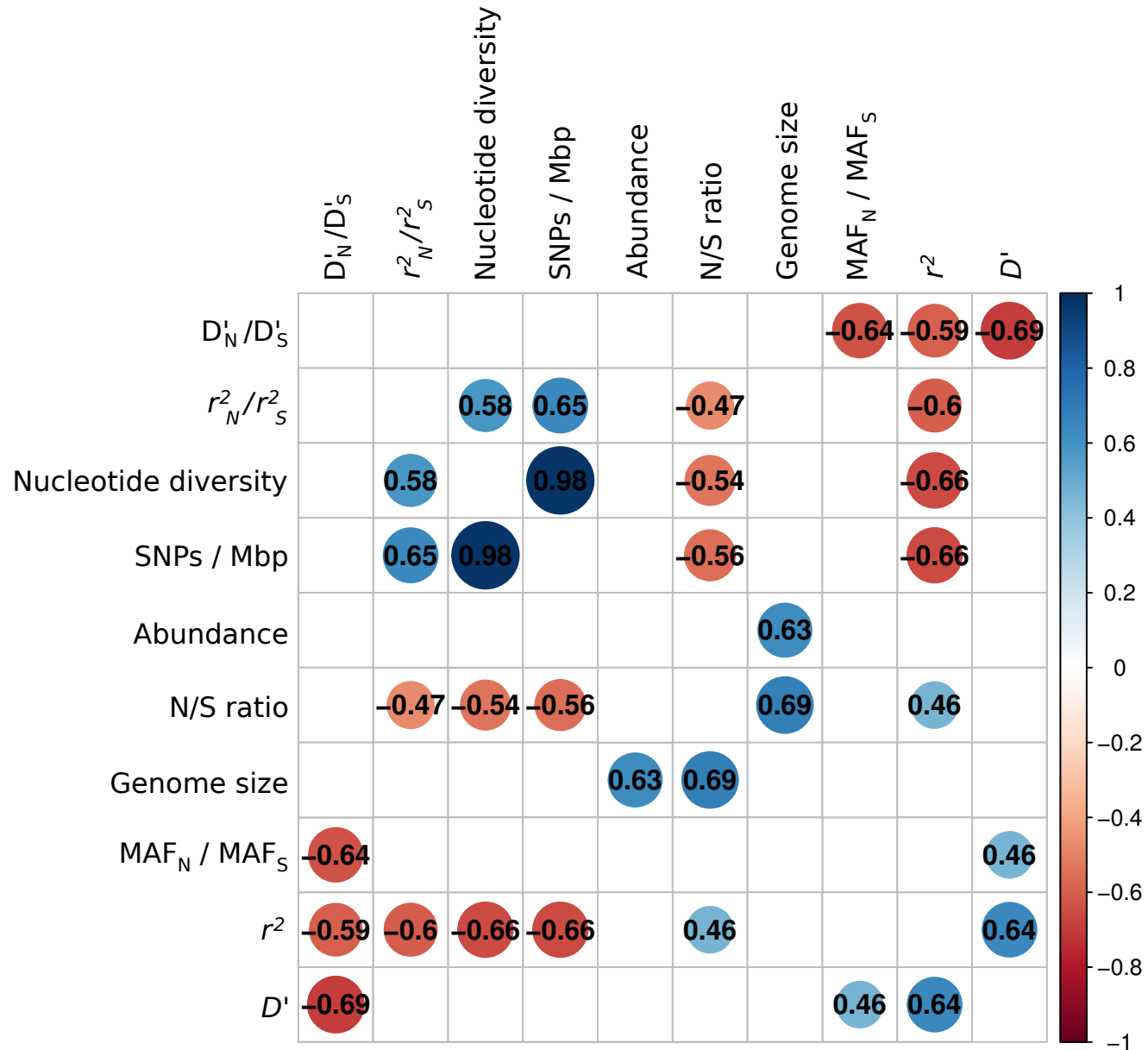

Supplement: Supplementary file 9 — Supplementary Figure S8 [file 41396_2020_655_MOESM9_ESM.pdf]

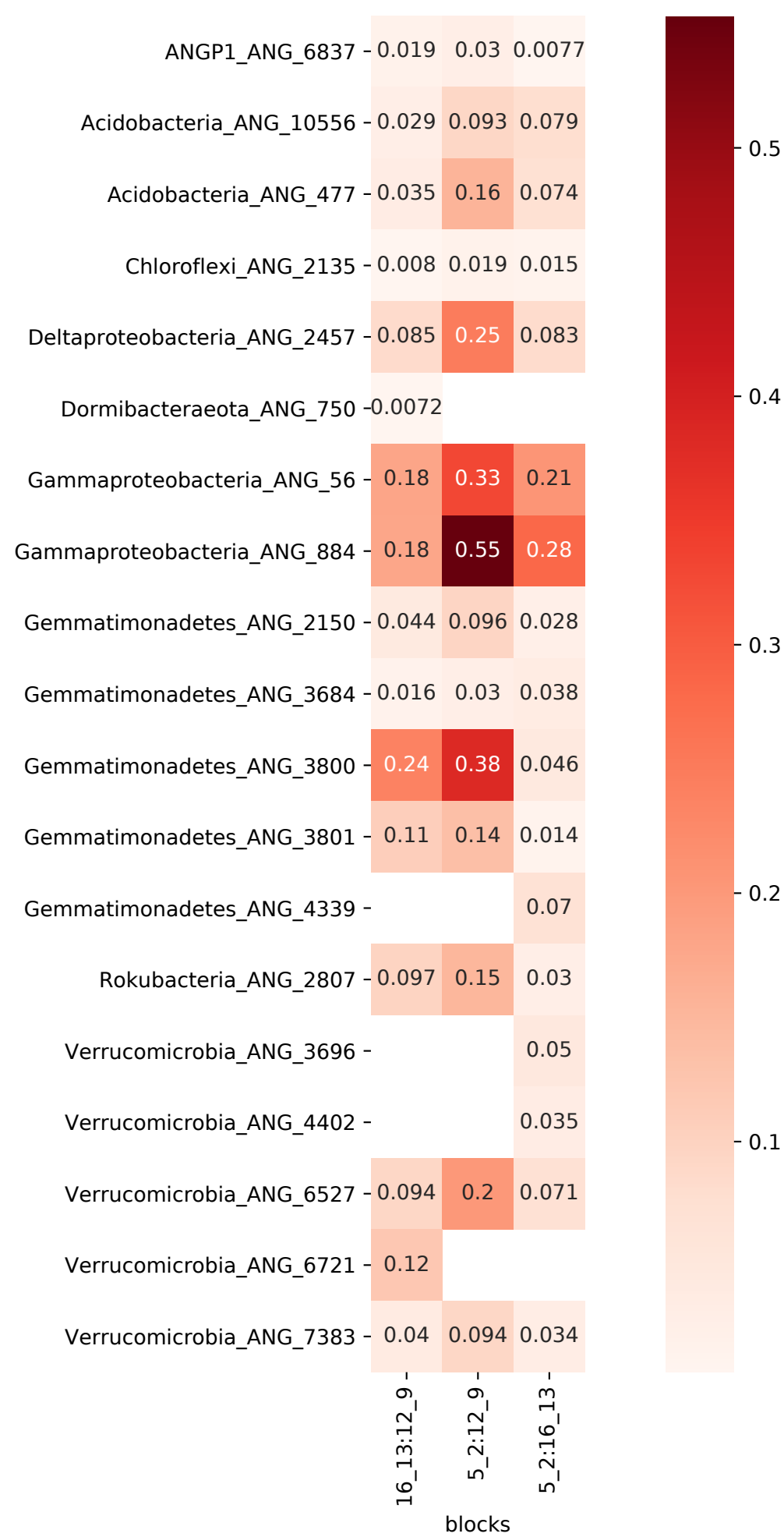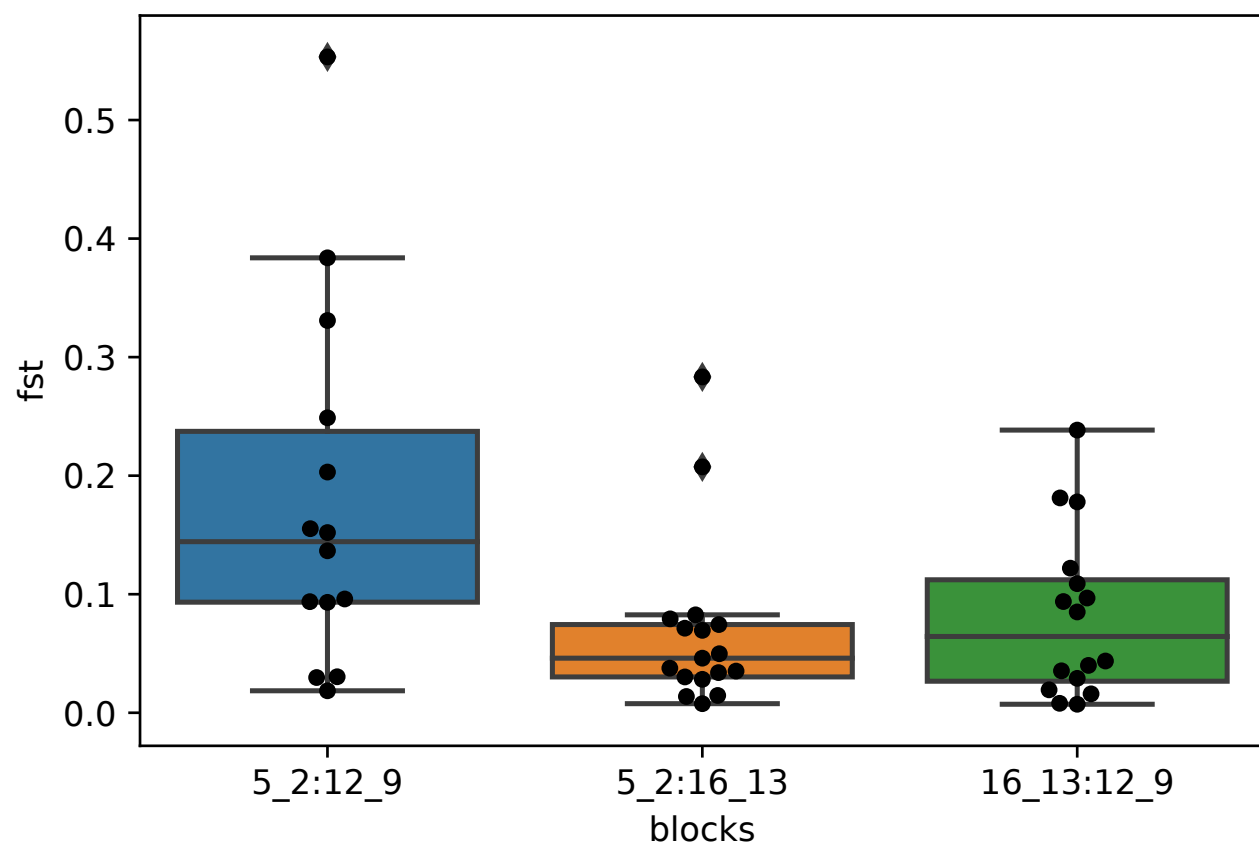

Supplement: Supplementary file 10 — Supplementary Figure S9 [file 41396_2020_655_MOESM10_ESM.pdf]

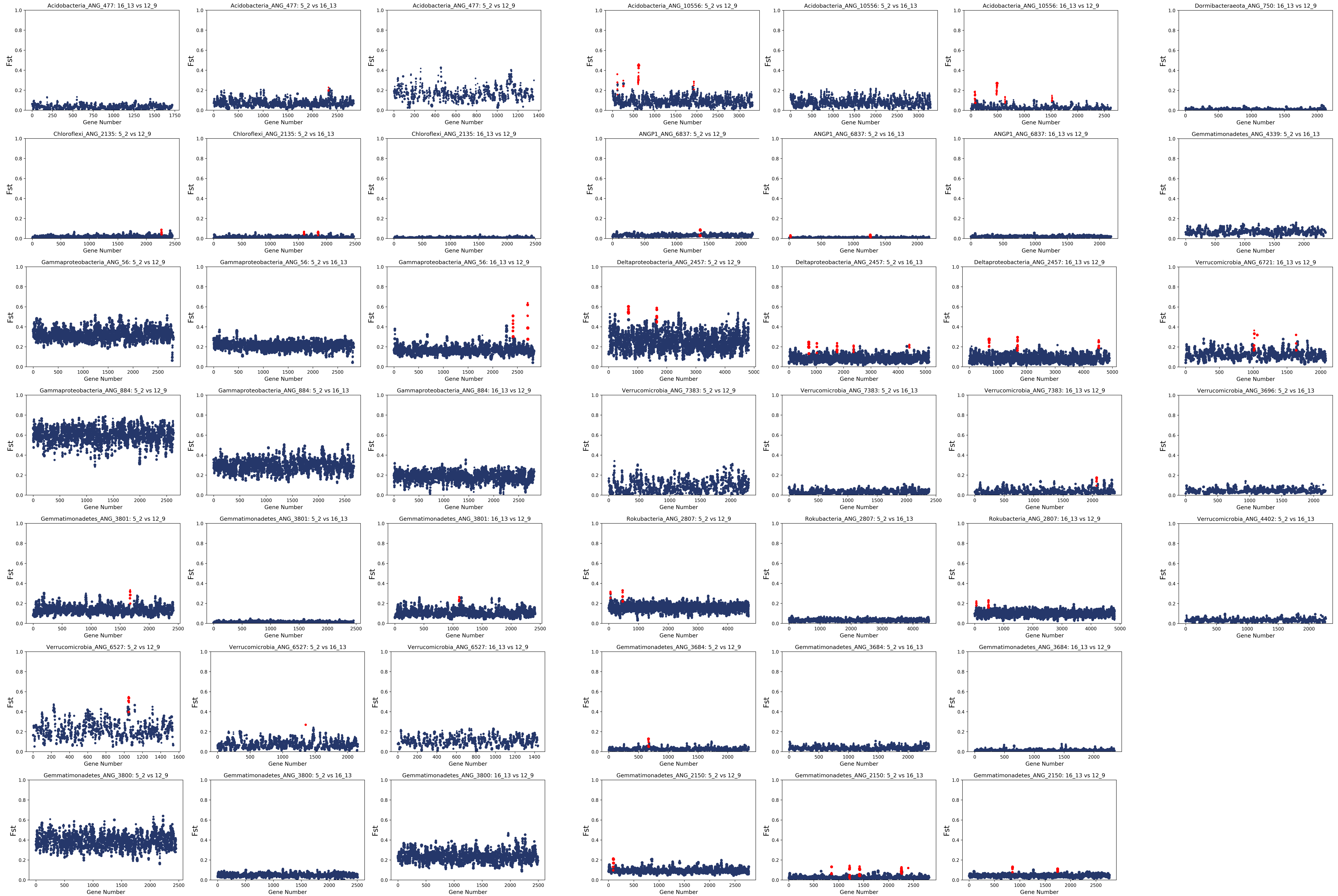

Supplement: Supplementary file 11 — Supplementary Figure S10 [file 41396_2020_655_MOESM11_ESM.pdf]
