# Supplementary material for: Soil bacterial populations are shaped by recombination and gene-specific selection across a grassland meadow
Source: ISME J. 2020 Apr 23;14(7):1834–46. doi: 10.1038/s41396-020-0655-x (PMC7305173; doi:10.1038/s41396-020-0655-x)
Supplement: Supplementary file 6 — Supplementary Figure S5 [file 41396_2020_655_MOESM6_ESM.pdf]

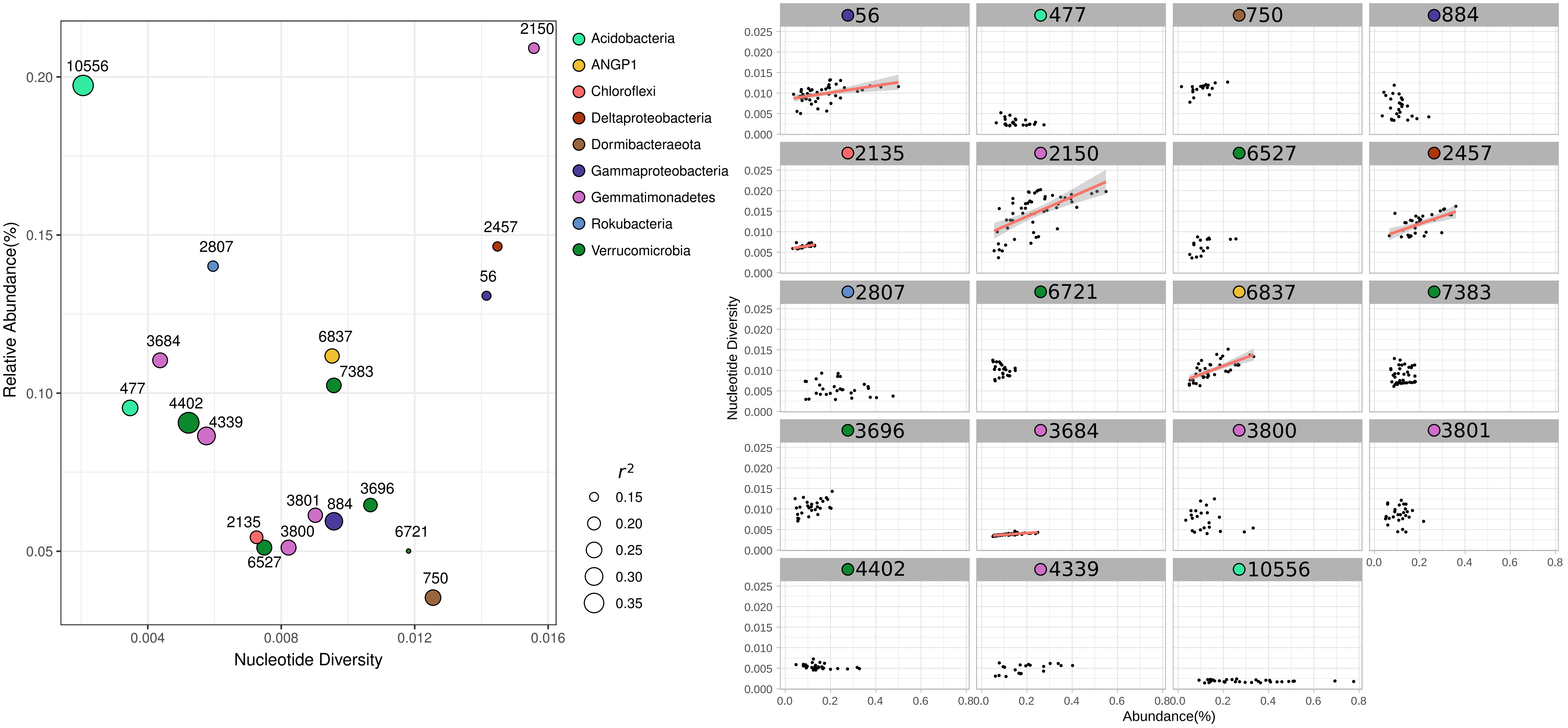

Relative Abundance(%)
Nucleotide Diversity
0.20
0.15
0.10
0.05
0.004
0.008
0.012
0.016
10556
2150
2807
2457
56
3684
6837
7383
477
4402
4339
3696
3801
884
2135
3800
6527
6721
750
Acidobacteria
ANGP1
Chloroflexi
Deltaproteobacteria
Dormibacteraeota
Gammaproteobacteria
Gemmatimonadetes
Rokubacteria
Verrucomicrobia
r^2
0.15
0.20
0.25
0.30
0.35
Nucleotide Diversity
Abundance(%)
56
477
750
884
2135
2150
6527
2457
2807
6721
6837
7383
3696
3684
3800
3801
4402
4339
10556
0.000
0.005
0.010
0.015
0.020
0.025
0.0
0.2
0.4
0.6
0.8
